# Ambient temperature and term birthweight in Latin American cities^☆^

**DOI:** 10.1016/j.envint.2022.107412

**Published:** 2022-09

**Authors:** Maryia Bakhtsiyarava, Ana Ortigoza, Brisa N. Sánchez, Ariela Braverman-Bronstein, Josiah L. Kephart, Santiago Rodríguez López, Jordan Rodríguez, Ana V. Diez Roux

**Affiliations:** a Institute of Urban and Regional Development, University of California Berkeley, USA; b Urban Health Collaborative, Dornsife School of Public Health, Drexel University, USA; c Department of Epidemiology and Biostatistics, Dornsife School of Public Health, Drexel University, USA; d Centro de Investigaciones y Estudios sobre Cultura y Sociedad, Consejo Nacional de Investigaciones Científicas y Técnicas y Universidad Nacional de Córdoba, Córdoba, Argentina; e Facultad de Ciencias Exactas, Físicas y Naturales, Universidad Nacional de Córdoba, Córdoba, Argentina

**Keywords:** Birthweight, Temperature, Latin America, Urban

## Abstract

**Background:**

Extreme temperatures may lead to adverse pregnancy and birth outcomes, including low birthweight. Studies on the impact of temperature on birthweight have been inconclusive due to methodological challenges related to operationalizing temperature exposure, the definitions of exposure windows, accounting for gestational age, and a limited geographic scope.

**Methods:**

We combined data on individual-level term live births (N≈15 million births) from urban areas in Brazil, Chile, and Mexico from 2010 to 2015 from the SALURBAL study (Urban Health in Latin America) with high-resolution daily air temperature data and computed average ambient temperature for every month of gestation for each newborn. Associations between full-term birthweight and average temperature during gestation were analyzed using multi-level distributed lag non-linear models that adjusted for newborn’s sex, season of conception, and calendar year of child’s birth; controlled for maternal age, education, partnership status, presence of previous births, and climate zone; and included a random term for the sub-city of mother’s residence.

**Findings:**

Higher temperatures during the entire gestation are associated with lower birthweight, particularly in Mexico and Brazil. The cumulative effect of temperature on birthweight is mostly driven by exposure to higher temperatures during months 7–9 of gestation. Higher maternal education can attenuate the temperature-birthweight associations.

**Interpretation:**

Our work shows that climate-health impacts are likely to be context- and place-specific and warrants research on temperature and birthweight in diverse climates to adequately anticipate global climate change. Given the high societal cost of suboptimal birthweight, public health efforts should be aimed at diminishing the detrimental effect of higher temperatures on birthweight.

**Funding:**

The Wellcome Trust.

## 1 Introduction

Birthweight is a function of various genetic, behavioral, socioeconomic, and environmental factors, and ambient temperature has been increasingly recognized as an environmental stressor associated with lower birthweight and other adverse birth outcomes (Chersich et al., 2020). The literature suggests that both hotter and colder temperatures during gestation can contribute to fetal oxidative stress and inflammation, which can reduce fetal growth and ultimately lead to lower birthweight (Ferguson et al., 2018, Halonen et al., 2010, Kahle et al., 2015). Despite a growing body of literature on the impacts of temperature on birthweight, the overall results are inconclusive (Chersich et al., 2020, Zhang et al., 2017, Strand et al., 2011). Some studies have shown that warmer temperatures, hot days, and higher temperature variability during pregnancy are associated with lower birthweight (Kloog et al., 2018, Li et al., 2018, Yitshak-Sade et al., 2020, Jakpor et al., 2020), while others have reported that colder temperatures are associated lower birthweight (Ngo and Horton, 2016, Ha et al., 2017), and still other studies found no clear relationship (Son et al., 2019).

Several factors may explain these divergent results. Only recently did studies begin to routinely consider nonlinearity in the temperature-birthweight relationship (Li et al., 2018, Yitshak-Sade et al., 2020, Jakpor et al., 2020, Yitshak-Sade et al., 2021), whereas the majority of studies have modeled the temperature-birthweight relationship linearly (Zhang et al., 2017). Most studies have used trimesters as the exposure window (Kloog et al., 2018, Ha et al., 2017, Basu et al., 2018, Bakhtsiyarava et al., 2018). However, there is currently no well-established theory on the length of exposure windows and timing of exposure most relevant to the temperature-birthweight relationship (Strand et al., 2011). Exposure measured by trimesters may introduce bias as fetal growth does not exactly follow trimesters (Wilson et al., 2017). Birthweight is a cumulative result of conditions during the entire gestational period, so it is necessary to consider exposure during the entire gestation. Analyses investigating each exposure window separately, as well as analyses where exposures covering the entire gestational period are specified together without an appropriate lag structure may result in incorrect identification of critical exposure windows (Wilson et al., 2017). In addition, while most recent studies explicitly account for the newborn’s gestational age, earlier studies did not (Beltran et al., 2014), thus conflating term newborns who weigh little due to fetal growth retardation with premature babies who weigh little in absolute terms but have weights in accordance with their gestational age. Finally, most of the work on temperature-birthweight is from single-site studies in the Global North, while evidence from lower- and middle-income regions – such as Latin America, where the prevalence of small term babies (in terms of weight or length for gestational age) is at 11% (Lee et al., 2013) – is sparse, preventing a holistic understanding of the relationship between temperature and birthweight.

We used unique data on all live births across 265 cities in three large countries in Latin America (Brazil, Mexico, and Chile) to describe the relationship between birthweight and ambient temperature at different points during gestation. We contribute to the prior work by (1) selecting the most appropriate form (linear vs non-linear) of the temperature-birthweight relationship; (2) expanding the growing but still limited literature on environmental effects on birth outcomes that uses fine-scale exposure windows; (3) using a large live births sample from Latin America -- an extremely understudied region of the world that is also characterized by different climatic patterns compared to the more researched regions of the Global North. Given the warming trends across Latin America, growing urban population, urban heat island effect, and high frequency of adverse pregnancy outcomes, this study provides important descriptive evidence regarding ambient temperature exposure and human wellbeing.

## 2 Methods

### 2.1 Data and measures

#### 2.1.1 Live births data

**Fig. 1:**
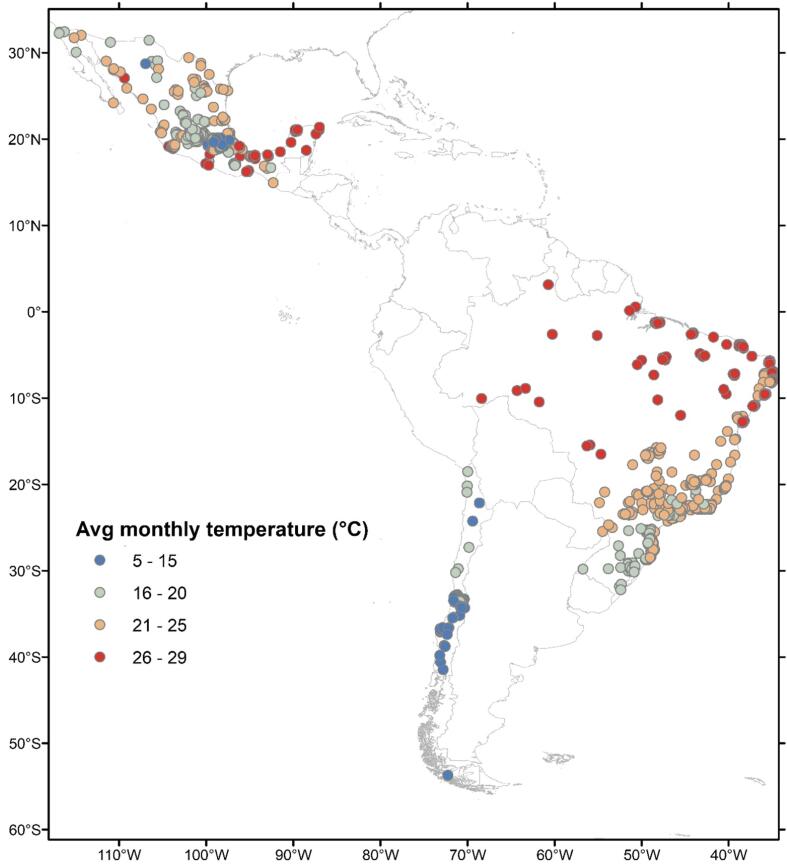
Map of average monthly temperature during 2010–2015 in 909 sub-cities of Brazil, Chile, and Mexico.

This study is part of the SALURBAL (Salud Urbana en América Latina/Urban Health in Latin America) project (Quistberg et al., 2019). SALURBAL is an interdisciplinary multinational collaboration aimed at investigating the social and environmental determinants of health in Latin American cities (Quistberg et al., 2019). As part of SALURBAL, we obtained the live births data from the live birth registries collected by the countries’ national statistical offices. Among the 11 SALURBAL countries, we included live births from Brazil, Mexico, and Chile as these countries had data on the day, month, and year of birth necessary for assigning temperature exposure. We focused on live births clustered in 909 sub-cities (Fig. 1). SALURBAL defines cities as clusters of administrative units that encompass the visually apparent urban extent or built up area of all urban agglomerations of > 100,000 residents (Quistberg et al., 2019). The administrative areas that compose cities (e.g., municipalities) are referred to as sub-cities (Quistberg et al., 2019).

The outcome is birthweight (in grams) for term births during 2010–2015. Informed by the existing literature, we extracted the following mother and child-level covariates that impact weight at birth: child’s sex, mother’s age (in years), education (primary, secondary, higher than secondary), partnership status (yes/no), and whether the mother had any previous births. Data on mother’s smoking status and weight/height were not registered in the vital statistics where the live births come from. Term births were defined as those 38–41 weeks of gestation according to the categorical variable of gestational age (<28 weeks; 28–37 weeks; 38–41 weeks; >41 weeks). After excluding live births with missing data on birthweight, the length of gestational age, and other covariates (Fig. S1 in Supplementary Material), the final sample consisted of 8,079,872 live births in Brazil; 6,405,777 live births in Mexico; and 890,156 live births in Chile. Overall, the level of missingness amounted to 0.2% in the case of Chile and 9% for both Brazil and Mexico.

#### 2.1.2 Temperature data

We used ambient temperature (at 2 m above land surface) data from the land surface component of the 5th generation of European ReAnalysis (ERA5), also known as ERA5-Land, produced by the European Centre for Medium-Range Weather Forecasts (ECMWF) (Muñoz, 2019). ERA5-Land is a global, publicly available reanalysis dataset that produces high-resolution (9x9 km) hourly meteorological data. Temperature data are described in more detail in Supplementary Material S2. We computed population-weighted mean daily atmospheric temperature for every sub-city for 2010–2015 using the gridded WorldPop population counts for 2010 available at 100 m × 100 m spatial resolution. The WorldPop data can be obtained here: https://www.worldpop.org/project/list. Weighting the temperature data by population distribution allowed us to obtain a more accurate measure of true temperature exposure compared to assigning temperature from the ERA5-Land grids to the entire population of a sub-city without taking into account population distribution. We also used the Köppen climate classification (Beck et al., 2018) that divides the Earth’s main climates into groups based on temperature and dryness, and further into subgroups by seasonal patterns of precipitation (Table S4 in Supplementary Material).

#### 2.1.3 Exposure windows

Term live births (38–41 weeks of gestation) were recoded as having occurred at 40 weeks of gestation to assign temperature exposure. Using the date of birth, we subtracted forty weeks from the date of birth to obtain the start date of gestation and, knowing the start and end dates, divided each gestational period into nine months or forty weeks. Then, we temporally and spatially linked the term live births data and temperature data at the sub-city level using mother’s sub-city of residence at the time of child’s birth and computed average ambient temperature for every month and week of gestation for each newborn.

### 2.2 Distributed lag models

We estimated multi-level distributed lag non-linear models (DLNMs) to analyze the relationship between ambient temperature and term birthweight. DLNMs have been widely used in temperature-mortality studies (Gasparrini, 2014) and only recently started being used in temperature-birthweight research (Jakpor et al., 2020, Yitshak-Sade et al., 2021). The DLNMs model a relationship between a time-varying exposure (average temperature at every month of gestation) and outcome measured a single time (birthweight). The DLNMs are particularly relevant when a health outcome measured at a particular time is the result of cumulative exposure over time – which is the case for birthweight. For every lag period, the lag-response component of the DLNMs models the non-linear association between temperature and birthweight in each exposure window (e.g., month of gestation) while adjusting for exposure that occurred over all the other months. This feature of the DLNMs is particularly advantageous for an analysis of temperature and birthweight because there is no unified theory on which period during gestation is most critical for the health of a newborn. The model also yields an estimate of the pregnancy-wide cumulative association between temperature and birthweight.

### 2.3 Model selection and estimation

To choose an optimal functional form of the temperature-birthweight association, we estimated a series of multi-level distributed lag models: (1) a model that estimated the relationship between temperature exposure and birthweight linearly; and (2) non-linear models with varied placement of the knots for modeling the non-linear temperature-birthweight association. Please refer to Supplementary Material S3 for model specification details for this step. After estimating these linear and non-linear models with different functional forms of the temperature-birthweight associations, we selected the final model as one with the overall best fit as indicated by smallest Akaike Information Criterion (AIC) (Gasparrini, 2014). A non-linear model was statistically preferred based on the AIC comparison. Therefore, the final model specified birthweight as a function of average temperature during each month of gestation with natural cubic splines with internal knots at the 10th, 25th, 50th, 75th, and 90th percentiles of the country-specific temperature, as favored by the AIC comparison approach and because of their wide use in the literature (Gasparrini et al., 2015). The lag-response curve was modeled with natural cubic splines with knots at months 1, 3, 5, 7 and 9 for every pregnancy.

For each term newborn we modelled term birthweight as a continuous outcome variable with temperature as the primary predictor described above. We estimated separate models for every country because the distribution of temperature and birthweight differed by country, which could result in country-specific patterns in the temperature-birthweight relationship. We adjusted for newborn’s sex, season of conception, calendar year of child’s birth, and controlled for the following maternal characteristics: categorical age, education, partnership status, presence of previous births, and climate zone. Births were clustered within sub-cities by including a random intercept for the sub-city of mother’s residence.

For displaying the results, we use 19 °C as a reference temperature—average temperature across all sub-cities in the study. The estimates relative to each country’s average temperature are reported in Supplementary Material Table S9 and Fig. S10. Having a single reference temperature enables us to compare associations within and between countries. For the cumulative associations, we estimate a difference in birthweight at a given country-specific temperature percentile (5th, 50th, 95th) compared to the reference temperature. For the monthly exposures, we estimate difference in birthweight associated with a 5-degree higher temperature in a given gestational month compared to the reference temperature (19 °C).

Models were also stratified by infant sex and by maternal education (separate stratifications) to examine the potential differential effects by these factors. Maternal education as an indicator of a mother’s socioeconomic position can be reflective of her access to and use of prenatal care, diet during pregnancy, and timing of fertility, all of which can impact birthweight (Silvestrin et al., 2013). Because of the small number of live births to mothers in the lowest education category (less than primary) we combined this category with live births to mothers who did not complete secondary education to facilitate the estimation of the stratified models. Since testing interactions of the non-linear temperature-birthweight associations and other factors is not straightforward in the non-linear distributed lag models, we instead examined whether the stratified models improve model fit. We compared the AIC of the primary model (where sex/education is modeled as a categorical predictor) to the sum of the AIC from the stratified models. When the AIC of the primary model is higher, stratification yields better fit. Model estimation was done using the “dlnm” package of the R Environment for Statistical Computing (Gasparrini et al., 2010).

### 2.4 Sensitivity checks

As mentioned above, we included live births within 38–41 weeks of gestation because of how the categorical variable on gestational age was coded. The data on gestational age for Mexico and Chile were available in weeks but it was not available for Brazil. We conducted a sensitivity analysis for Mexico and Chile as these countries had data on gestational age in weeks and included births at 37, 39, 40, 41, and 42 weeks. The DLNMs require that all live births have the same time lags in the model structure, which precluded us from estimating a pooled model with varying gestational age. To deal with that complication, we estimated models using 10 months (42 weeks) as the main exposure period; for those births occurring before 42 weeks we assigned exposure in the missing weeks using temperature from the last observed week. This sensitivity analysis is similar to that in Jakpor et al. (Jakpor et al., 2020).

We also conducted the following sensitivity checks: an analysis for nulliparous mothers; an analysis based on the continuous variable of gestational age (available for Mexico and Chile); modeling average temperature for every month during the three-month preconception period, in addition to the main gestation period; repeated the main analysis for live births stratified by climate zone and the patterns of inter-annual temperature variability.

## 3 Results

### 3.1 Sample characteristics

Our samples represent 422 sub-cities in Brazil, 406 in Mexico, and 81 in Chile. The average term birthweight among the countries varied from about 3200 to 3400 g (Table 1). Most mothers in the sample were younger than 30 years old, and slightly less than a half of them were first-time mothers. Educational attainment and partnership status differed substantially across the countries. In Chile, 77.5% of live births were to women who at least completed secondary education, compared with 21.3% in Brazil, where we also observed the highest percentage of live births to women with primary and uncompleted secondary education – 20.5%. The majority of live births in Mexico were to women who had a partner or those in a stable relationship (88.3%), compared to 50.8% in Brazil and only 30.1% in Chile. Temperature-wise, sub-cities in Chile were the coolest in the sample (Fig. 1), while sub-cities in Mexico and Brazil had greater variability in climatic conditions (Table S4 in Supplementary Material).

**Table 1:** Summary statistics of the term live births samples during 2010–2015 used in the analysis.

Variable	**Brazil**		**Mexico**		**Chile**	
	Mean or %	SD	Mean or %	SD	Mean or %	SD
Total number of live births	8,079,872		6,405,777		890,156	
Number of sub-cities	422		406		81	
Number of cities	152		92		21	
Number of live births per sub-city	19,147	53,677	15,778	24,569	10,990	7,840
*Outcome*						
Birthweight (g)	3,252	451	3,235	377	3,435	417
*Exposure*						
Average monthly temperature during gestation (°C)	22.2	2.9	18.9	4.0	14.0	2.4
*Covariates*						
Sex of infant						
Male	51.0%		51.4%		50.6%	
Female	49.0%		48.6%		49.4%	
*Mother’s characteristics*						
Age						
<25 years	39.7%		49.9%		36.1%	
25–29 years	25.5%		24.7%		24.9%	
30–34 years	21.5%		16.4%		22.4%	
≥ 35 years	13.5%		9.05%		16.6%	
Education						
Primary and uncompleted secondary	20.5%		5.0%		2.9%	
Completed primary; uncompleted secondary	58.2%		56.7%		19.7%	
Secondary and above	21.3%		38.3%		77.5%	
Had previous births	55.2%		58.3%		54.3%	
In a stable relationship at the time of birth^1^	50.8%		88.3%		30.1%	
Season of conception^2^						
Winter	26.7%		26.3%		25.5%	
Spring	24.6%		24.4%		24.5%	
Summer	23.6%		23.9%		24.4%	
Fall	25.1%		25.4%		4%25.5%	
Year of birth						
2010	17.2%		16.0%		17.0%	
2011	16.3%		16.8%		16.8%	
2012	15.8%		17.0%		16.5%	
2013	16.2%		17.0%		16.4%	
2014	17.0%		16.9%		16.9%	
2015	17.5%		16.4%		16.4%	

1 In Mexico, this variable included women in “union libre” (an arrangement similar to the U.S.’ common law marriage), whereas in Chile and Brazil only married women were included.

2 Hemisphere-specific seasons.

**Fig. 2:**
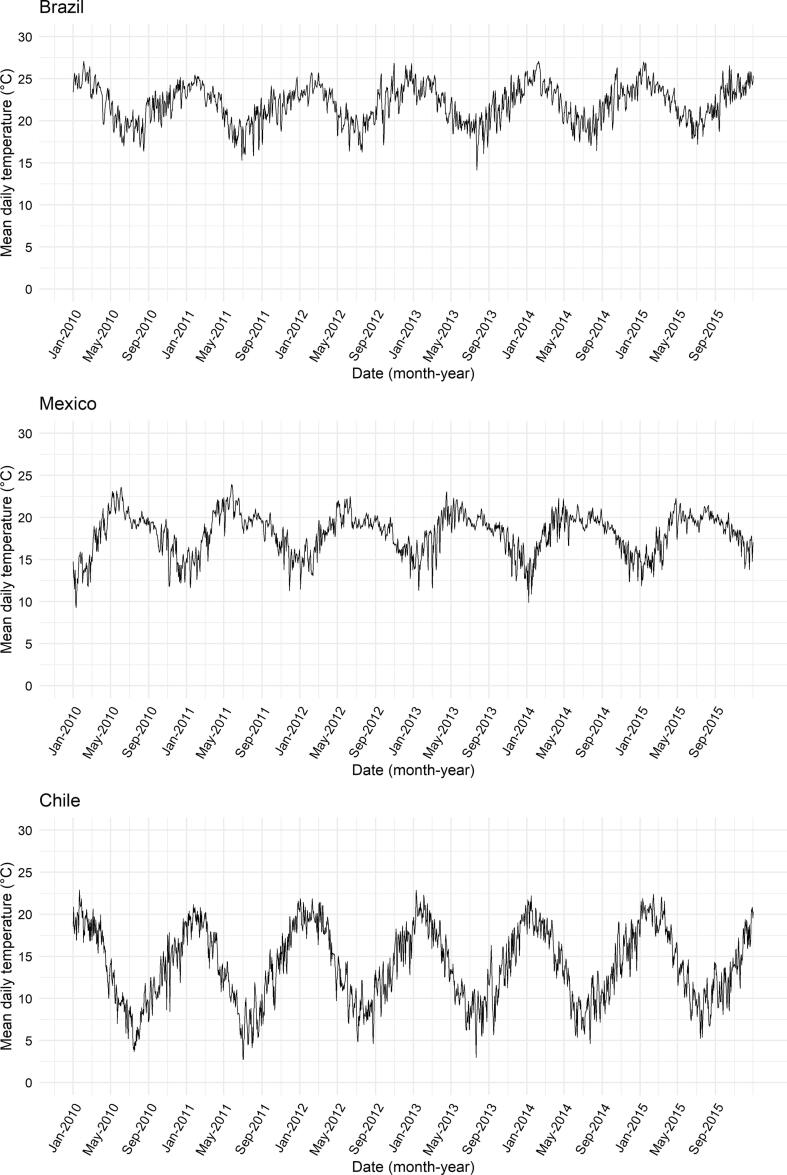
Seasonal patterns of sub-city daily mean temperature in Brazil, Mexico, and Chile during the 2010–2015 study period.

As Fig. 2 shows, the seasonal patterns of temperature differed across countries. The daily mean temperature in Brazil was 22.0 °C, the highest in the sample, followed by 18.1 °C in Mexico and 13.9 °C in Chile. The average annual difference between maximum and minimum daily temperature (temperature range) during 2010–2015 was the highest in Chile at 19.9 °C, followed by 15.1 °C in Mexico, and 13.7 °C in Brazil. Chile, the coldest country, exhibited the largest fluctuations in daily temperature across seasons.

### 3.2 Cumulative associations

**Fig. 3:**
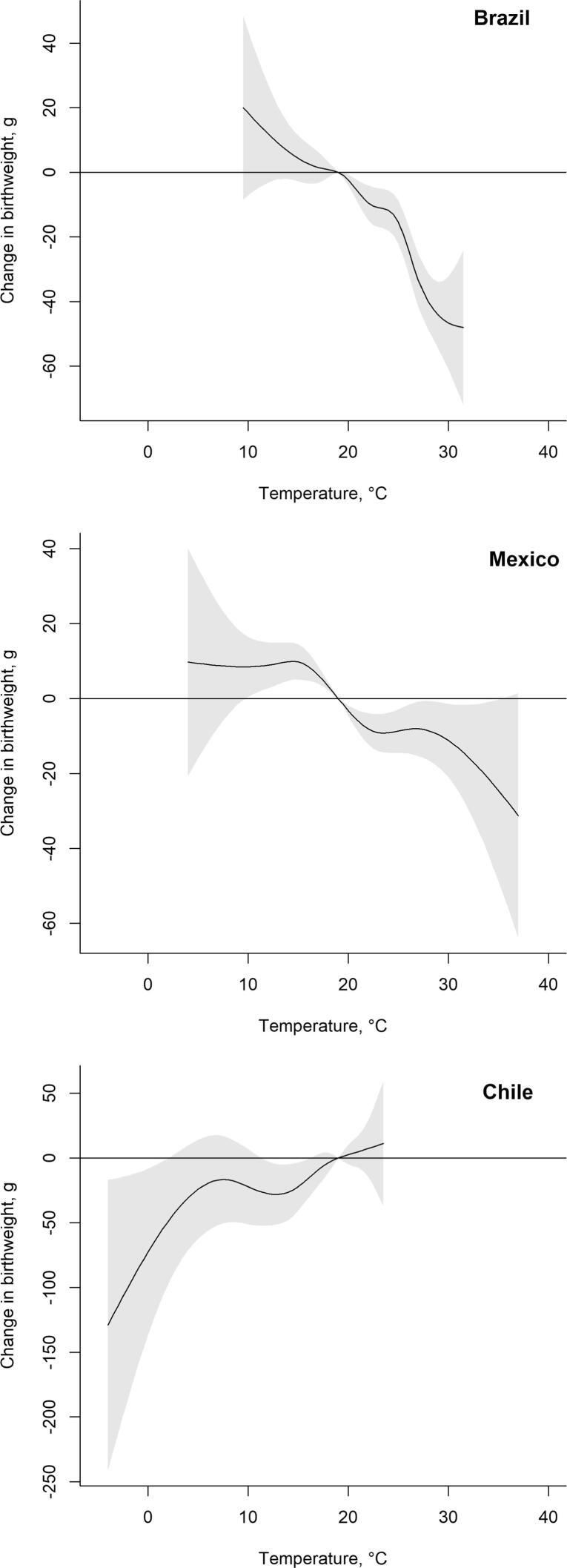
Cumulative associations between average monthly temperature during nine months of gestation and term birthweight for term newborns in 2010–2015. The curves depict an estimated difference in birthweight associated with average temperature during gestation relative to a reference temperature of 19 °C (average monthly temperature across the countries). The curves are derived from distributed lag non-linear models adjusted for child sex, mother’s age, education, partnership status, whether she had previous births, calendar year of child’s birth, season of conception, climate zone, and include random intercepts for the sub-city of mother’s residence at the time of the child’s birth (Tables S5-S7 in Supplementary Material). Temperature on the x-axis refers to the average monthly temperature during the entire gestation. The estimates relative to each country’s average temperature are reported in Supplementary Material Table S9.

Fig. 3 and Table 2 present cumulative associations between average monthly temperature during gestation and term birthweight, adjusted for the child- and mother-level covariates. In Table 2, point estimates reflect the mean difference in term birthweight associated with gestation-average temperatures in the 5th, 50th, and 95th percentiles of country-specific temperature, compared to 19 °C. In Brazil, gestation period average temperatures below 19 °C were associated with higher birthweight whereas temperatures above 19 °C were associated with lower birthweight, although not all associations were statistically significant: for example, a gestation average temperature in the 50th percentile (22.3 °C) was associated with a 10.41-gram (95% CI: −16.28; −4.54) lower birthweight, compared to 19 °C. We observe a similar pattern for Mexico except that associations of lower temperature with higher birthweight are statistically significant: relative to a gestation-average of 19 °C, gestation-average temperature in the 5th percentile of Mexico’s temperature distribution (p50 = 11.9 °C) is associated with an 8.87-gram (95% CI: 2.72; 15.01) higher birthweight. For most of the temperature range in Chile we do not observe statistically significant associations, though point estimates suggest that lower temperatures (below 19 °C) are associated with lower birthweight. A gestation-average temperature in the 50th percentile of Chile temperature distribution (p50 = 14.2 °C) is associated with 26.38-gram lower birthweight (95% CI: −47.92; −4.85), compared to the 19 °C-reference. In sum, for the cumulative associations, average monthly temperatures during gestation that are higher than 19 °C are generally associated with lower birthweight, whereas most estimates for the temperatures lower than the reference (19 °C) are not statistically significant.

**Table 2:** Mean differences in birthweight associated with cumulative exposure to average temperature in the 5th, 50th and 95th percentiles of country-specific temperature distribution, compared to a reference temperature of 19 °C among term live births in 2010–2015.

	Percentiles of the temperature distribution		
Country	5th percentile	50th percentile	95th percentile
Brazil	*16.0 °C*2.67 (−3.46; 8.80)	*22.3 °C* −10.41 (−16.28–4.54)	*27.4 °C* −36.99 (−45.50; −28.49)
Mexico	*11.9 °C*8.87 (2.72; 15.01)	*18 °C*3.28 (2.17; 4.39)	*28 °C* −8.55 (−16.43; −0.68)
Chile	*6.5 °C* −17.98 (−53.62; 17.65)	*14.2 °C* −26.38 (−47.92; −4.85)	*21.2 °C*4.85 (−7.55; 17.25)

Estimates in grams (95% Confidence Interval in parentheses). Cumulative associations between average monthly temperature during nine months of gestation and birthweight for term newborns in 2010–2015. Estimates derived from the distributed lag non-linear models presented in Fig. 3. Temperature in the top row refers to the percentile-specific temperature for that country. An equivalent table with estimates derived relative to country-specific average temperatures is available in Supplementary Material Table S9.

### 3.3 Monthly exposure windows

**Fig. 4:**
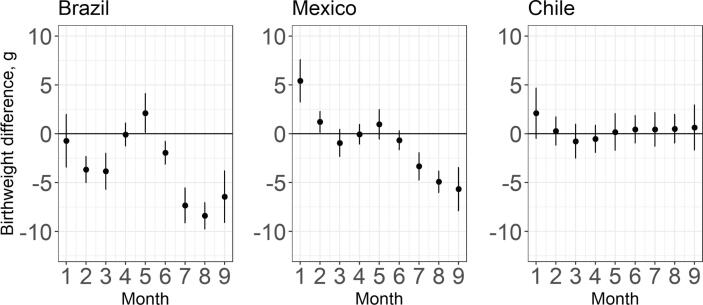
Difference in mean birthweight (with 95% CI) associated with a 5 °C higher temperature in each month of gestation relative to a 19 °C gestation average (average across the countries) among term newborns in 2010–2015. The estimates are obtained from distributed lag non-linear models and adjusted for child sex, mother’s age, education, partnership status, whether the mother had previous births, calendar year of child’s birth, season of conception, climate zone, and include a random intercept for the sub-city of mother’s residence at the time of the child’s birth. Estimates for every exposure window account for temperature exposure during all the other exposure windows during the gestational period. Numerical estimates and the confidence intervals from the figure are provided in Supplementary Material Table S8. An equivalent figure with estimates derived relative to country-specific average temperatures is available in Supplementary Material Fig. S10.

Fig. 4 depicts differences in mean birthweight associated with a 5 °C higher temperature in each month of gestation, relative to the average temperature of 19 °C. In Brazil, a 5 °C higher ambient temperature (relative to an average of 19 °C) is associated with lower birthweight in months 2 and 3 of gestation, and in months 6–9. In Brazil, the birthweight difference associated with exposure to 5 °C higher temperature than the reference varies from −1.95 g (95% CI −3.11; −0.79) in month 6 to −8.4 g (95% CI −9.74; −7.06) in month 8. In Mexico, higher temperatures during the first two months of gestation are associated with higher birthweight, but toward the end of gestation, particularly, in months 7 through 9, higher temperatures are associated with lower birthweight. The magnitude of these month-specific associations in Mexico is similar to that in Brazil. No statistically significant associations are observed in Chile, which is the coldest country in our sample (Fig. 4).

### 3.4 Analyses by strata: child’s sex and mother’s education

We did not observe a marked difference in the temperature-birthweight relationship between the sexes (Supplementary Fig. S11), and the stratification by sex did not improve the fit of the models for any of the countries.

**Fig. 5:**
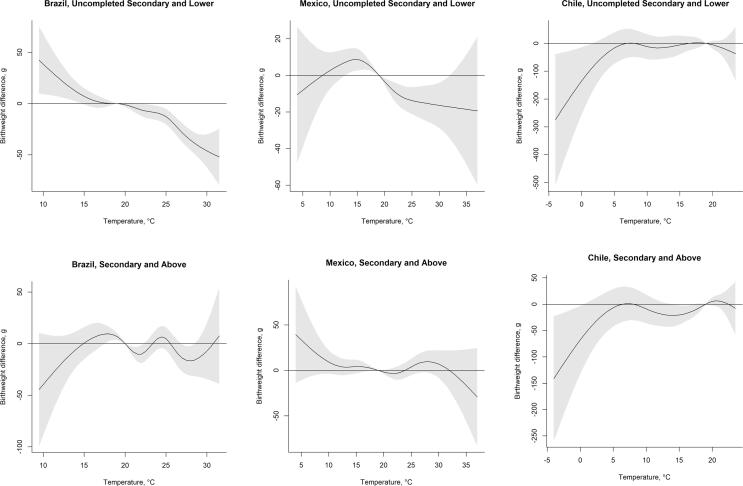
Difference in birthweight associated with a 5 °C higher temperature in each month of gestation, relative to a 19 °C gestation average (average across the countries), stratified by maternal education. See Table 3 for technical details.

In contrast, maternal education appears to modify the temperature-birthweight association. Stratifying by maternal education improved model fit and yielded different estimates of the temperature-birthweight association across education strata. For the highest temperatures, we observe associations between temperature and birthweight that are larger in magnitude among the women in the lowest educational strata (Table 3 and Fig. 5). For example, in Brazil (Table 3) term newborns born to women with uncompleted secondary education or lower have, on average, 32.36-gram lower birthweight (95% CI: −41.93; –22.78) associated with exposure to average temperature during gestation in the 95th percentile of Brazil’s monthly temperature distribution relative to the reference temperature of 19 °C, whereas for mothers with at least secondary education the estimate is –23.13 (95% CI: −38.26; −8.00). In Mexico, exposure to gestation-average temperatures in the 95th percentile of Mexico’s temperature distribution (28 °C) is associated with 15.44-gram lower (95% CI: −25.40; −5.47) birthweight among the term live births to mothers with uncompleted secondary education or lower. For those born to mothers with at least secondary education in Mexico the estimate is positive at 9.63 g (95% CI: −2.42; 21.68) though not statistically significant. In line with the main results for Chile, we observe mostly non-statistically significant associations between temperature and birthweight among the women in the two education strata.

**Table 3:** Mean differences in birthweight associated with cumulative exposure to average temperature in the 5th, 50th and 95th percentiles of country-specific temperature distribution, compared to a reference temperature of 19 °C among term live births in 2010–2015 stratified by maternal education.

		Percentiles of the temperature distribution		
Country	Education	5th percentile	50th percentile	95th percentile
Brazil		*16.0 °C*	*22.3 °C*	*27.4 °C*
	Uncompleted secondary and lower	3.46 (−3.45; 10.37)	−7.24 (−13.89; −0.58)	–32.36 (−41.93; –22.78)
	Secondary and above	−1.66 (−14.36; 11.04)	−14.56 (−26.16; −2.96)	–23.13 (−38.26; −8.00)
Mexico		*11.9 °C*	*18 °C*	*28 °C*
	Uncompleted secondary and lower	5.99 (−1.72; 13.70)	3.16 (1.74; 4.58)	−15.44 (−25.40; −5.47)
	Secondary and above	4.43 (−5.25; 14.11)	1.81 (0.07; 3.55)	9.63 (−2.42; 21.68)
Chile		*6.5 °C*	*14.2 °C*	*21.2 °C*
	Uncompleted secondary and lower	−1.77 (−55.04; 51.49)	−10.15 (−48.75; 28.44)	−12.25 (−34.8; 10.30)
	Secondary and above	0.19 (–33.10; 33.48)	−14.35 (−42.70; −0.01)	6.12 (−5.98; 18.22)

Estimates in grams (95% Confidence Interval in parentheses). Cumulative associations between average monthly temperature during the nine months of gestation and birthweight for term newborns in 2010–2015 stratified by maternal education. The estimates are obtained from distributed lag non-linear models stratified by maternal education and adjusted for child sex, mother’s age, partnership status, whether the mother had previous births, calendar year of child’s birth, season of conception, climate zone, and include a random intercept for the sub-city of mother’s residence at the time of the child’s birth.

The results of the sensitivity analyses described in the methods section are presented in Figs. S12-S23 in the Supplementary material. The results in the main text were robust to the sensitivity analyses.

## 4 Discussion

### 4.1 Main findings

We analyzed associations between average monthly ambient temperature during gestation and term birthweight. Our results show that higher temperatures (relative to the 19 °C reference) during gestation are associated with lower birthweight, particularly in Mexico and Brazil, after accounting for newborn and maternal characteristics, seasonality, and climate zone. This potential cumulative effect of warmer temperatures on birthweight is mostly driven by stronger temperature-birthweight associations during the last months of gestation. The associations between higher temperatures and lower birthweight were of greater magnitude during months 7–9 of gestation, particularly in Brazil and Mexico, where warmer temperatures are more common. We found no sex differences in the temperature-birthweight association, but maternal education may modify the association – the results suggest that higher levels of maternal education attenuate the negative temperature-birthweight association observed especially at very high temperatures (95th percentiles of country-specific average monthly temperatures).

Evident from the estimates for Brazil and Mexico, we mostly observe statistically significant negative associations between temperature and birthweight for warm (in absolute terms) temperatures. Chile is the coldest country in our sample, so one potential explanation for the lack of statistically significant associations between higher temperatures and birthweight in Chile is that air temperature there does not reach levels high enough to trigger a physiological response affecting birthweight. The absence of statistically significant negative associations between lower-than-average temperatures and birthweight compared to the statistically significant estimates observed for average temperatures (Table 2) may suggest adaptation to cooler temperatures in Chile. Moreover, babies in Chile might be better protected from the detrimental effect of warm temperatures given the high levels of access to prenatal medical care and overall better socioeconomic conditions evidenced by lower maternal and infant mortality and higher human development index, compared to Mexico and Brazil. (United Nations Children’s Fund, 2021) This finding emphasizes the importance of considering location-specific climate impacts and the need to conduct analyses of temperature-birthweight relationships in diverse climates.

### 4.2 Public health implications

Most of the predicted differences in birthweight associated with temperature range between approximately −40 to +11 g. While not large from an individual-level perspective, these differences may decrease the average birthweight at the population level and lead to a higher prevalence of term low birthweight. We estimate that, should the average monthly temperature increase by 5 °C in Mexico relative to its current levels, the average birthweight would decrease by 13 g. The same temperature increase (5 °C) in Brazil and Chile would be associated with a 15-gram and 11-gram decrease in average birthweight, respectively. In addition to these public health considerations, studies have shown that a 10% increase in birthweight is associated with a 0.9% increase in earnings and 1.2% increase in high school graduation (Black et al., 2007). Therefore, lower birthweight associated with temperature can have negative economic and public health consequences.

### 4.3 Comparison with other studies

Our findings of higher temperatures associated with lower birthweight and critical exposure windows at the end of gestation are consistent with other work (Ha et al., 2017, Basu et al., 2018). For example, studies from the U.S. by Ha et al. (2017) and Yitshak-Sade et al. (2021) found that high temperatures during the last trimester or the last few weeks of gestation are associated with lower birthweight. Our results identified critical exposure windows in months 7–9 of pregnancy, which agrees with these studies, even though they assessed the relationship using different exposure windows (trimesters and weeks). The nonlinearity of the temperature-birthweight association is also in line with results from previous work. Li et al. found that, compared to maternal exposure to maximum temperature above 30 °C, exposure to 20–25 °C was associated with 11-g (95% CI 8;18) higher birthweight (Li et al., 2018). Similarly, Yitshak-Sade et al. (2021) revealed a non-linear association between average weekly temperature during gestation and birthweight, with the temperature effect sustained at the end of gestation.

It should be noted that none of these prior studies were conducted in Latin America, so we are not comparing studies from areas with similar political, socioeconomic, and climatic conditions. Moreover, because our study relied on distributed lag models, which so far have been rarely used in this line of work, our results and the results from other studies using different methods are not directly comparable. One exception is a recent study by Jakpor et al. (2020) on a cohort of term births in France, which showed that weather variability (standard deviation) of weekly temperature during pregnancy is associated with decreases in birthweight, while mean weekly temperature is not. Like our study, Jakpor et al. used distributed lag models, but the AIC comparison approach in their case favored a linear form of the temperature-birthweight relationship, and they also focused on a shorter timescale – weeks. Another study that utilized the same class of models (distributed lag nonlinear) is a U.S.-based study by Yitshak-Sade et al. (2021) discussed above.

### 4.4 Biological pathways potentially involved in the temperature-birthweight association

The etiological pathway linking temperature exposure during gestation and birthweight has not been agreed upon, but several mechanisms could be at play. Pregnant women have a limited ability to thermoregulate because of the increased metabolic demands of pregnancy. Weight gain, increased fat deposition, and the decreasing ratio of surface area to body mass can increase core body temperature and reduce pregnant women’s ability to lose heat through sweating, which makes them vulnerable to hot temperatures, heat shocks, and concomitant acute fetal distress (Wells and Cole, 2002). In addition, heat stress can trigger the production of heat-shock proteins that have been associated with preeclampsia and preterm birth, which are closely related to fetal growth retardation and low birthweight, respectively (Beltran et al., 2014). Another potential explanation is the rise in inflammatory, hemostatic, and lipid markers during heat exposure that could lead to hypertension and diabetes during pregnancy and eventually to lower birthweight (Wells and Cole, 2002). Overall, the literature suggests that any inflammatory pathway associated with non-optimal temperatures could trigger fetal growth retardation and ultimately lower birthweight.

### 4.5 Influence of maternal education and newborn sex in the temperature-birthweight association

Our finding of no difference in the temperature-birthweight association among male and female newborns is unlike the results from the two most recent studies (Jakpor et al., 2020, Yitshak-Sade et al., 2021). However, these studies are from different locations than ours – USA and France, and, while Jakpor et al. (2020) found females to sustain greater negative impacts from higher temperatures, Yitshak-Sade et al. (2020) found males to be more vulnerable. It should be noted that while Yitshak-Sade et al. (2020) used average weekly temperature during gestation, the results from Jakpor et al. (2020) concern differential vulnerability to temperature variation, not mean temperature, among male and female infants. More evidence is needed to make a conclusive statement regarding differential susceptibility among male and female newborns. Though not many studies analyzed temperature-birthweight association by mother’s education, our finding of greater susceptibility to warmer temperatures for mothers with low education is in line with other work (Yitshak-Sade et al., 2021). Mothers with higher educational attainment may have better resources for prenatal care, nutrition, and living/working conditions, which are recognized protective factors against low birthweight. In addition, higher maternal education is closely related to a better socioeconomic status and adequate living conditions, which might minimize exposure to heat during pregnancy.

### 4.6 Strengths

Our study is innovative in several ways. First, the use of distributed lag non-linear models allowed us to specify the most appropriate and data-driven functional form of the temperature-birthweight relationship. The lagged structure of the exposure–response association allowed us to identify critical exposure windows during gestation while accounting for exposure in the other months. This is an important contribution given that (1) only recently did studies routinely begin to consider non-linearity and the lagged structure of exposure–response in studies of temperature and birthweight (Li et al., 2018, Yitshak-Sade et al., 2020, Jakpor et al., 2020); (2) the existing studies have been heterogeneous with respect to whether they model temperature exposure over the entire gestation or only focus on selected periods (Li et al., 2018, Basu et al., 2018). Birthweight is a cumulative result of conditions during the entire gestation, so focusing on select time periods – as opposed to exposure windows that cover the entire gestational period – may provide an incomplete picture of the temperature conditions relevant to birthweight. Second, this study is one of the few multi-site studies of temperature and birthweight. By including multiple cities with different climatic conditions, our analysis presents more robust evidence for the association between temperature and birthweight. Third, this study also extends the geographic scope of our knowledge on birthweight. So far, most studies have focused on countries in the Global North and some countries in sub-Saharan Africa. Latin America is extremely underrepresented in this research; two recent reviews of epidemiologic evidence on the association between temperature and pregnancy outcomes did not include/identify a single study on temperature and birthweight from Latin America (Chersich et al., 2020, Zhang et al., 2017). By focusing on a large sample of live births from multiple cities in Latin America, a region where an estimated 11% of births measure or weigh too small for their gestational age (Lee et al., 2013), we address the issue of limited knowledge on the temperature-birthweight relationship in climates other than those in the Global North – such as tropical and temperate climates with different temperature and precipitation patterns.

## 5 Limitations

Our study is not without limitations. First, the temperature exposure variables are ecological and represent an approximation of women’s actual exposure to temperature, which is in turn determined by how much time they spend outside, their activity patterns, and the use of air conditioning inside. As such, exposure misclassification could be present in the study. We suspect this potential misclassification to be non-differential as ambient temperature at 2 m above surface is assigned to women independent of factors that could modify their actual exposure (e.g., the use of air conditioning). Relatedly, temperature exposure measured at sub-city level or another spatial unit (as opposed to measuring it at women’s residential address) is also conceptually appropriate if one thinks of potential population-level interventions (e.g., those that address climate change or other area-level interventions like greening) to minimize exposure to heat. Second, we did not have information on mothers’ moves to other addresses prior to giving birth. However, three factors may alleviate the implications of the absence of this information on the results: 1) On average, residential mobility during pregnancy is often local and is unlikely to result in a drastically different temperature exposure (Bell and Belanger, 2012); 2) Most women do not move, though an estimated 9–32% may move (Bell and Belanger, 2012, Chen et al., 2010); 3) New residential location is unlikely chosen on its temperature regime, so the misclassification error is non-differential. Research on the impact of residential mobility during pregnancy on the measurement of air pollution exposure (we did not identify studies that used temperature as exposure) concluded that the results from studies that did not consider residential mobility during pregnancy were robust to that omission (Warren et al., 2018). The third limitation refers to how gestational age was measured. In Brazil and Chile, gestational age was determined based on 1) women’s last menstrual period or 2) the 1st trimester ultrasound if the data on the last menstrual period was not available. For Mexico gestational age was based on the Capurro exam. Early-pregnancy ultrasound is currently the most accurate way to determine gestational age, and pregnancy dating based on the recall of the last menstrual period or a clinical assessment of newborns tend to overestimate gestational age (Lee et al., 2017). As such, weekly exposure windows based on the gestational age data in our sample would likely be off by a few weeks, with implications for the accuracy of temperature exposure. Monthly exposure windows may be more robust to this misspecification. Relatedly, the continuous gestational age variable was available for Mexico and Chile but not for Brazil, and we relied on the categorical variable in the main text for consistency. Fifth, while air pollution is associated with lower birthweight (Yitshak-Sade et al., 2021), we did not control for it because temperature plays a role in the formation and quantity of air pollutants (Buckley et al., 2014, Reid et al., 2012); therefore air pollution may be on the causal path between temperature and birthweight (Jakpor et al., 2020). Sixth, data on whether the live births were a result of multiple gestation were not available. According to the estimates of the rates of multiple gestation in the countries of interest, as much as 10% of our sample may represent non-singleton births (Smits et al., 2011). We were also not able to collect information about mother’s health behavior pre- and during pregnancy, such as smoking status, BMI, alcohol drinking habits, diabetes, or hypertension, which can contribute to fetal growth retardation. The study may lose power due to not adjusting for these important predictors of birthweight. However, not adjusting for these factors is unlikely to result in confounding bias. Conditioning on term births may have created collider bias. We adjusted for common causes of prematurity and low birthweight including education, age and birth order but cannot rule out the possibility of collider bias. Finally, our conclusions can only be applied to live births as our study did not consider outcomes such as still births, miscarriages, and abortions.

## 6 Conclusions

We find that warmer temperatures during gestation may be associated with lower birthweight, yet it appears that higher maternal education can buffer the effects. Given the high societal and economic cost of suboptimal birthweight as reflected by worse health outcomes later in life, lower incomes, and lower educational attainment, public health efforts should be aimed at diminishing the potential detrimental effect of higher temperatures on birthweight.

## Declaration of Competing Interest

The authors declare that they have no known competing financial interests or personal relationships that could have appeared to influence the work reported in this paper.

## Data Availability

Live births data for Brazil, Chile, and Mexico were obtained from statistical agencies in each country. A link to these agency websites can be accessed via https://drexel.edu/lac/data-evidence/data-acknowledgements/. Temperature data is publicly available at https://cds.climate.copernicus.eu/cdsapp#!/dataset/reanalysis-era5-single-levels?tab=overview. **Code availability** Requests for the code used in this study should be made to the corresponding author.
