# Supplementary material for: Ambient temperature and term birthweight in Latin American cities ☆
Source: Environ Int. Author manuscript; Available in PMC 2022 Sep 7. (PMC9376808; doi:10.1016/j.envint.2022.107412)
Supplement: Supplementary data 1 [file EMS151806-supplement-Supplementary_data_1.docx]

**SUPPLEMENTARY MATERIAL**


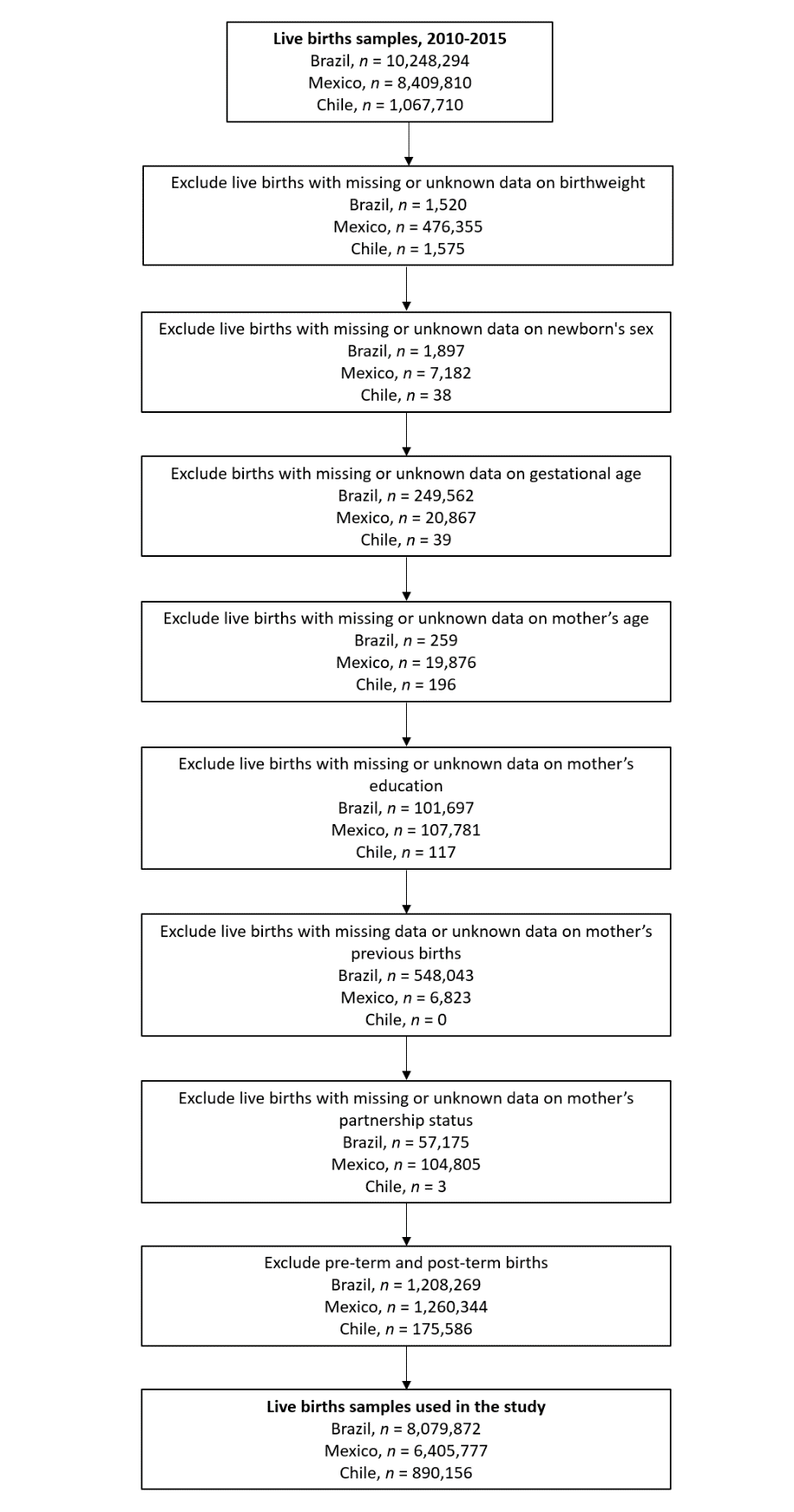


Figure S1. Study flowchart

S2: Imputation details

ERA5-Land is a land surface dataset, and it ignores pixels that have more than 50% coverage by water,^1^ resulting in missing data in coastal cities. To fill in the missing temperature pixels for parts of those coastal cities, we used another dataset produced by the ECMWF – ERA5^2^ – to impute the missing values. An average of 3.68 % of all ERA5-Land pixels were imputed in Brazil, 0.56% in Mexico, and 1.48% in Chile.

The imputation was carried out as follows. First, we resampled the hourly ERA5 data from a spatial resolution of 31 x 31 km to match the 9 x 9 km resolution of the ERA5-Land data. Second, we built the following random forest model to impute the missing values for each day and each geographic unit with missing pixels:

$$T_{ERA5-Land}=f\left( X \right)+ e (Eq.1)$$

In Equation 1, $T_{ERA5-Land}$ is the imputed hourly temperature value; X is a vector of variables that includes: (1) ERA5 hourly data resampled from 31 km to 9 km resolution; (2) absolute elevation; (3) relative elevation, obtained as the difference in elevation between a 9x9 km pixel and its surrounding; (4) aspect. The residual term *e* was modeled with kriging spatial interpolation.

To avoid overfitting, we used cross-validation to tune the parameters of the random forest and spatial kriging. We ran the model using all pixels within a unit’s geographic boundary and within a 15-pixel buffer around the boundary. Overall, an average of 3.68 % of all ERA5-Land pixels were imputed in Brazil (80 out of 422 sub-cities had imputed pixels), 0.56% in Mexico (37 out of 406 sub-cities were affected), and 1.48% in Chile (19 out of 81 sub-cities were affected).

S3: Model selection and estimation

To choose an optimal functional form of the temperature-birthweight association, we estimated a series of multi-level distributed lag models: 1) linear model; 2) non-linear model where the temperature-birthweight association is modeled with natural cubic splines with internal knots at the 10^th^, 25^th^, 50^th^, 75^th^, and 90^th^ percentiles of the country-specific temperature distribution; 3) non-linear model with natural cubic splines with internal knots at 10^th^, 50^th^, and 90^th^ percentiles; 4) non-linear model with natural cubic splines with internal knots at 25^th^, 50^th^, and 75^th^ percentiles. We chose the primary model based on the minimum Akaike Information Criterion (AIC).^3^

For a secondary analysis based on weekly exposure windows (results not shown) we estimated multi-level non-linear distributed lag models with continuous birthweight specified as a function of average temperature during each week of gestation with natural cubic splines with internal knots at the 10^th^ 25^th^, 50^th^, 75^th^, and 90^th^ percentiles of the country-specific temperature. The lag-response curve was modeled with natural cubic splines with knots at weeks 1, 14, 27 and 40 for every gestation period. Births were clustered within sub-cities by including a random intercept for the sub-city of mother’s residence.

Table S4: The number of live births and sub-cities per climate zone

|  | **Brazil** | | | **Mexico** | | **Chile** | | |
| --- | --- | --- | --- | --- | --- | --- | --- | --- |
| Climate zone | N (live births) (%) | | N (sub-cities) | N (live births) (%) | N (sub-cities) | N (live births) (%) | | N (sub-cities) |
| Tropical rainforest | 573,095 (7.1%) | | 18 |  |  |  | |  |
| Tropical monsoon | 596,922 (7.4%) | | 27 | 199,060 (3.1%) | 20 |  |  |  |
| Dry tropical savanna | 768,693 (9.5%) | | 49 | 110,876 (1.7%) | 5 |  |  |  |
| Wet tropical savanna | 2,860,152 (35.4%) | | 151 | 646,884 (10.1%) | 41 |  |  |  |
| Hot arid steppe | 45,154 (0.6%) | | 2 | 1,443,630 (22.5%) | 63 |  |  |  |
| Cold arid steppe |  |  |  | 630,463 (9.8%) | 52 | 293,557 (33.0%) | | 26 |
| Hot arid desert |  |  |  | 523,144 (8.2%) | 17 |  |  |  |
| Cold arid desert |  |  |  |  |  | 140,099 (15.7%) | | 8 |
| Temperate without dry season, hot summer | 2,857,816 (35.4%) | | 153 | 89,541 (1.4%) | 4 |  |  |  |
| Temperate without dry season, cold summer | 270,364 (3.3%) | | 12 | 67,106 (1%) | 11 | 42,280 (4.7%) | | 3 |
| Temperate dry summer, hot summer |  |  |  | 16,425 (0.3%) | 3 |  |  |  |
| Temperate dry summer, warm summer |  |  |  | 98,844 (1.5%) | 2 | 402,011 (45.2%) | | 42 |
| Temperate dry winter, hot summer | 80,158 (1%) | | 7 | 503,653 (7.9%) | 36 |  |  |  |
| Temperate dry winter, warm summer | 27,518 (0.3%) | | 3 | 20,72,473 (32.4%) | 151 |  |  |  |
| Temperate dry winter, cold summer |  |  |  | 3,678 (0.1%) | 1 |  |  |  |
| Polar tundra |  |  |  |  |  | 12,209 (1.4%) | | 2 |
| Total number of live births | 8,079,872 | | | 6,405,777  406 |  | 890,156 | | |
| Number of sub-cities | 422 | | |  |  | 81 | | |

Table S5. Results from the multi-level distributed lag non-linear models for Brazil estimating an association between average temperature during gestation and birthweight^1^

| Variable | Estimate | SE^3^ | 95% CI^3^ |
| --- | --- | --- | --- |
| *Child and mother covariates^2^* |  |  |  |
| Sex of infant (male) | 117.153 | 0.312 | 116.541, 117.765 |
| *Mother's age (ref=mothers <25 y.o.)* |  |  |  |
| 25-29 years | 23.683 | 0.418 | 22.864, 24.502 |
| 30-34 years | 23.667 | 0.461 | 22.763, 24.571 |
| ≥ 35 years | 6.476 | 0.542 | 5.414, 7.538 |
| *Mother’s education (ref=primary and uncompleted secondary)* |  |  |  |
| Less than primary | -34.111 | 0.414 | -34.922, -33.30 |
| Completed secondary and above | -11.424 | 0.429 | -12.265, -10.583 |
| Mother had previous births | 81.053 | 0.346 | 80.375, 81.731 |
| Mother in a stable relationship at the time of birth | 13.113 | 0.343 | 12.441, 13.785 |
| *Year of birth (ref=2010)* |  |  |  |
| 2011 | -3.845 | 0.585 | -4.992, -2.698 |
| 2012 | 8.234 | 0.603 | 7.052, 9.416 |
| 2013 | 7.049 | 0.560 | 5.951, 8.147 |
| 2014 | 14.240 | 0.554 | 13.154, 15.326 |
| 2015 | 17.511 | 0.572 | 16.39, 18.632 |
| *Season of conception (ref=Winter)* |  |  |  |
| Fall | 4.910 | 0.618 | 3.699, 6.121 |
| Spring | 1.620 | 0.639 | 0.368, 2.872 |
| Summer | 5.625 | 0.725 | 4.204, 7.046 |
| *Climate zone (ref=tropical rainforest)* |  |  |  |
| Tropical monsoon | 24.207 | 12.568 | -0.426, 48.840 |
| Tropical savanna, dry | 46.125 | 11.397 | 23.787, 68.463 |
| Tropical savanna, wet | -19.997 | 10.369 | -40.320, 0.326 |
| Arid steppe, hot | 21.139 | 30.640 | -38.915, 81.193 |
| Temperate without dry season, hot summer | -5.820 | 10.598 | -26.592, 14.952 |
| Temperate without dry season, cold summer | -42.991 | 15.830 | -74.018, -11.964 |
| Temperate dry winter, hot summer | -78.546 | 18.562 | -114.928, -42.164 |
| Temperate dry winter, warm summer | -89.071 | 25.913 | -139.86, -38.282 |
| *Model statistics* |  |  |  |
| Variance (sub-city) | 1,678  121,428,713  8,079,872  422 | | |
| AIC |  |  |  |
| N (live births) |  |  |  |
| N (sub-cities) |  |  |  |

1. Because temperature was modeled non-linearly, results for the temperature are presented as graphs in the main text. Temperature was modeled as average monthly temperature during every month of gestation. The model specification included a cross-basis function with natural cubic splines with knots at the 10^th^, 25^th^, 50^th^, 75^th^, and 90^th^ percentiles of the country-specific temperature distribution. The lag-response curve was also modeled with natural cubic splines with knots located at months 1, 3, 5, 7, and 9 of gestation.
2. The analysis focused on term births as defined by a categorical variable of gestational age (38-41 weeks), which we averaged to 40 weeks to compute exposure.
3. SE stands for Standard Error; 95% CI is 95% Confidence Interval.

Table S6. Results from the multi-level distributed lag non-linear models for Mexico estimating an association between average temperature during gestation and birthweight^1^

| Variable | Estimate | SE | 95% CI |
| --- | --- | --- | --- |
| *Child and mother covariates* |  |  |  |
| Sex of infant (male) | 68.220 | 0.290 | 67.656, 68.792 |
| *Mother's age (ref=mothers <25 y.o.)* | 35.076 | 0.378 | 34.335, 35.817 |
| 25-29 years | 45.725 | 0.441 | 44.861, 46.589 |
| 30-34 years | 47.463 | 0.550 | 46.385, 48.541 |
| ≥ 35 years |  |  |  |
| *Mother’s education (ref=primary and uncompleted secondary)* |  |  |  |
| Less than primary | -16.627 | 0.683 | -17.966, -15.288 |
| Completed secondary and above | 0.611 | 0.321 | -0.018, 1.240 |
| Mother had previous births | 34.435 | 0.327 | 33.794, 35.076 |
| Mother in a stable relationship at the time of birth | 12.503 | 0.460 | 11.601, 13.405 |
| *Year of birth (ref=2010)* |  |  |  |
| 2011 | -2.798 | 0.538 | -3.852, -1.744 |
| 2012 | -3.193 | 0.536 | -4.244, -2.142 |
| 2013 | -11.445 | 0.554 | -12.531, -10.359 |
| 2014 | -12.721 | 0.530 | -13.76, -11.682 |
| 2015 | -12.56 | 0.535 | -13.609, -11.511 |
| *Season of conception (ref=Winter)* |  |  |  |
| Fall | -0.802 | 0.635 | -2.047, 0.443 |
| Spring | 3.701 | 0.635 | 2.456, 4.946 |
| Summer | 2.996 | 0.643 | 1.736, 4.256 |
| *Climate zone (ref=tropical monsoon)* |  |  |  |
| Tropical savanna, dry | 64.151 | 22.779 | 19.504, 108.798 |
| Tropical savanna, wet | -7.659 | 12.486 | -32.132, 16.814 |
| Arid steppe, hot | 6.881 | 11.811 | -16.269, 30.031 |
| Arid steppe, cold | -91.223 | 12.417 | -115.56, -66.886 |
| Arid desert, hot | 79.914 | 15.085 | 50.347, 109.481 |
| Temperate without dry season, hot summer | 34.508 | 25.074 | -14.637, 83.653 |
| Temperate without dry season, cold summer | -92.207 | 17.558 | -126.621, -57.793 |
| Temperate dry summer, hot summer | 30.122 | 28.286 | -25.319, 85.563 |
| Temperate dry summer, warm summer | -39.408 | 33.708 | -105.476, 26.66 |
| Temperate dry winter, hot summer | -53.585 | 13.043 | -79.149, -28.021 |
| Temperate dry winter, warm summer | -124.016 | 11.354 | -146.27, -101.762 |
| Temperate dry winter, cold summer | -164.446 | 46.898 | -256.366, -72.526 |
| *Model statistics* |  | | |
| Variance (sub-city) | 2,042  93,823,341  6,405,777 | | |
| AIC |  |  |  |
| N (live births) |  |  |  |
| N (sub-cities) | 406 | | |

1. See notes for table S5 for technical details.

Table S7. Results from the multi-level distributed lag non-linear models for Chile estimating an association between average temperature during gestation and birthweight^1^

| Variable | Estimate | SE | 95% CI |
| --- | --- | --- | --- |
| *Child and mother covariates* |  |  |  |
| Sex of infant (male) | 111.416 | 0.867 | 109.717, 113.115 |
| *Mother's age (ref=mothers <25 y.o.)* |  |  |  |
| 25-29 years | 16.859 | 1.219 | 14.47, 19.248 |
| 30-34 years | 8.141 | 1.331 | 5.532, 10.75 |
| ≥ 35 years | -8.388 | 1.473 | -11.275, -5.501 |
| *Mother’s education (ref=primary and uncompleted secondary)* |  |  |  |
| Less than primary | 0.826 | 2.751 | -4.566, 6.218 |
| Completed secondary and above | -20.249 | 1.158 | -22.519, -17.979 |
| Mother had previous births | 89.795 | 0.985 | 87.864, 91.726 |
| Mother in a stable relationship at the time of birth | -14.391 | 1.044 | -16.437, -12.345 |
| *Year of birth (ref=2010)* |  |  |  |
| 2011 | -2.085 | 1.610 | -5.241, 1.071 |
| 2012 | -0.544 | 1.697 | -3.870, 2.782 |
| 2013 | -2.019 | 1.64 | -5.233, 1.195 |
| 2014 | 1.604 | 1.589 | -1.510, 4.718 |
| 2015 | -7.261 | 1.914 | -11.012, -3.510 |
| *Season of conception (ref=Winter)* |  |  |  |
| Fall | 4.910 | 0.618 | 3.699, 6.121 |
| Spring | 1.620 | 0.639 | 0.368, 2.872 |
| Summer | 5.625 | 0.725 | 4.204, 7.046 |
| *Climate zone (ref=cold arid steppe)* |  |  |  |
| Arid desert, cold | -13.922 | 14.514 | -42.369, 14.525 |
| Temperate without dry season, cold summer | 38.563 | 22.135 | -4.822, 81.948 |
| Temperate dry summer, warm summer | 9.352 | 8.963 | -8.215, 26.919 |
| Polar tundra | 1.110 | 28.405 | -54.564, 56.784 |
| *Model statistics* |  |  |  |
| Variance (sub-city) | 1,218  13,233,231  890,156  81 | | |
| AIC |  |  |  |
| N (live births) |  |  |  |
| N (sub-cities) |  |  |  |

1. See notes for table S5 for technical details

Table S8. Difference in mean birthweight (with 95% confidence interval) associated with a 5°C higher temperature in each month of gestation relative to a 19°C gestation average (average across the countries) among term newborns in 2010-2015. The estimates are obtained from distributed lag non-linear models and adjusted for child sex, mother’s age, education, partnership status, whether the mother had previous births, calendar year of child’s birth, season of conception, climate zone, and include a random intercept for the sub-city of mother’s residence at the time of the child’s birth. Estimates for every exposure window account for temperature exposure during all the other exposure windows during the gestational period.

| Country | Month of gestation | Estimate | 95% CI |
| --- | --- | --- | --- |
| Brazil | 1 | -0.724 | -3.404 1.957 |
|  | 2 | -3.678 | -5.011 -2.346 |
|  | 3 | -3.844 | -5.683 -2.004 |
|  | 4 | -0.084 | -1.253 1.085 |
|  | 5 | 2.120 | 0.126 4.115 |
|  | 6 | -1.947 | -3.109 -0.786 |
|  | 7 | -7.332 | -9.096 -5.569 |
|  | 8 | -8.396 | -9.737 -7.055 |
|  | 9 | -6.432 | -9.068 -3.797 |
| Mexico | 1 | 5.407 | 3.242 7.573 |
|  | 2 | 1.217 | 0.147 2.287 |
|  | 3 | -0.952 | -2.338 0.433 |
|  | 4 | -0.066 | -1.061 0.93 |
|  | 5 | 0.961 | -0.555 2.476 |
|  | 6 | -0.672 | -1.643 0.3 |
|  | 7 | -3.344 | -4.756 -1.933 |
|  | 8 | -4.929 | -6.025 -3.833 |
|  | 9 | -5.673 | -7.887 -3.459 |
| Chile | 1 | 2.104 | -0.464 4.672 |
|  | 2 | 0.266 | -1.177 1.708 |
|  | 3 | -0.773 | -2.507 0.961 |
|  | 4 | -0.534 | -1.93 0.862 |
|  | 5 | 0.166 | -1.707 2.039 |
|  | 6 | 0.445 | -0.955 1.845 |
|  | 7 | 0.451 | -1.273 2.174 |
|  | 8 | 0.510 | -0.937 1.957 |
|  | 9 | 0.635 | -1.679 2.95 |

Table S9. Mean differences in birthweight associated with cumulative exposure to average temperature in the 5^th^ and 95^th^ percentiles of country-specific temperature distribution, compared to country-specific average^1^ temperature among term live births in 2010-2015

|  | Percentiles of the temperature distribution | |
| --- | --- | --- |
| Country | 5^th^ percentile | 95^th^ percentile |
| Brazil | *16.0°C*  12.57 (4.76; 20.37) | *27.4°C*  -27.10 (-33.65; -20.54) |
| Mexico | *11.9°C*  8.87 (2.72; 15.01) | *28°C*  -8.55 (-16.43; -0.68) |
| Chile | *6.5°C*  8.41 (-17.49; 34.29) | *21.2°C*  31.24 (4.87; 57.60) |

Estimates in grams (95% CI). Cumulative associations between average monthly temperature during the entire gestational period (nine months) and birthweight for term newborns in 2010-2015. Estimates derived from the distributed lag non-linear models described in Figure 3 in the main text. Temperature in the top row refers to the percentile-specific temperature for that country.

^1^Average monthly temperature in Brazil is 22.2°C; 18.9°C in Mexico, and 14.0°C in Chile.


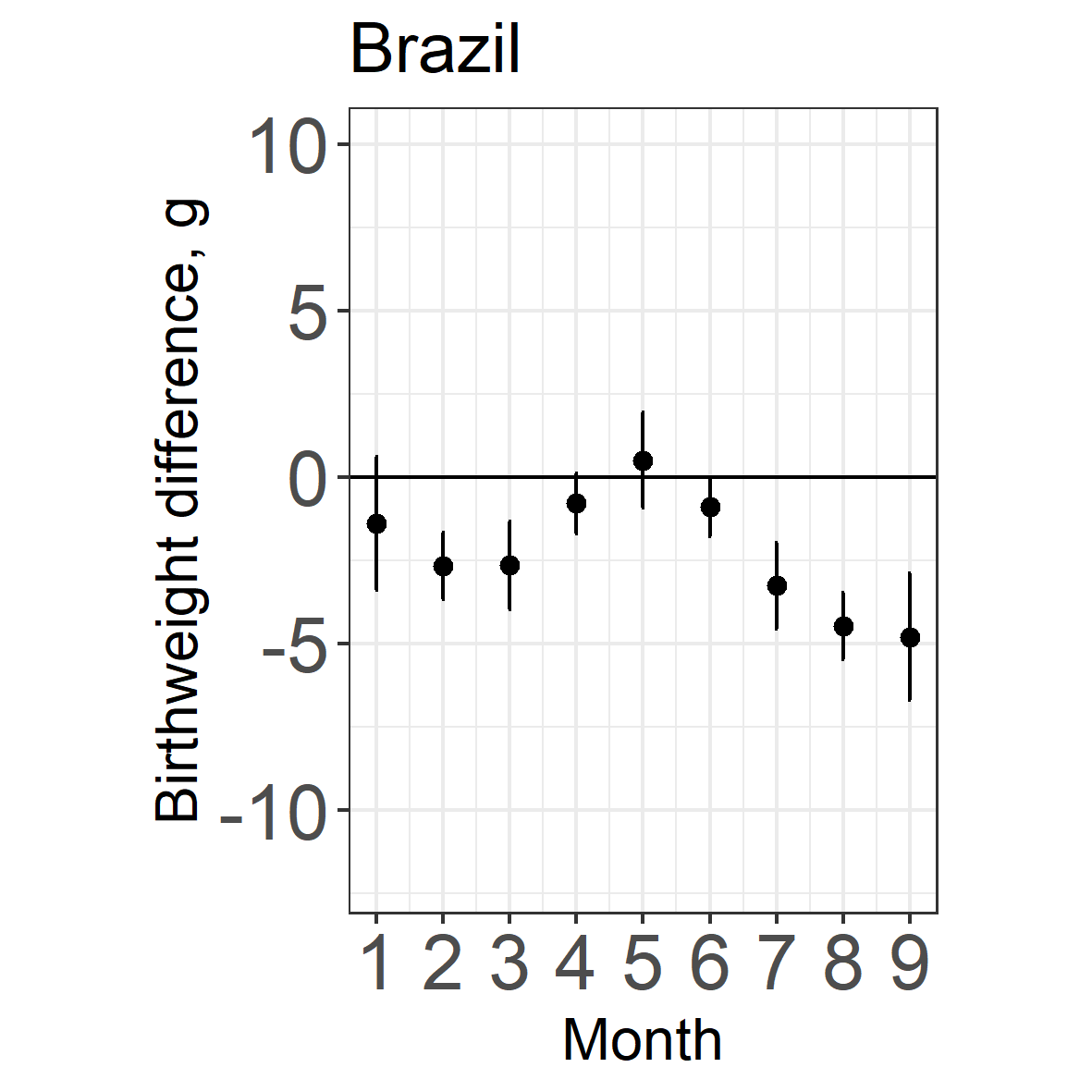

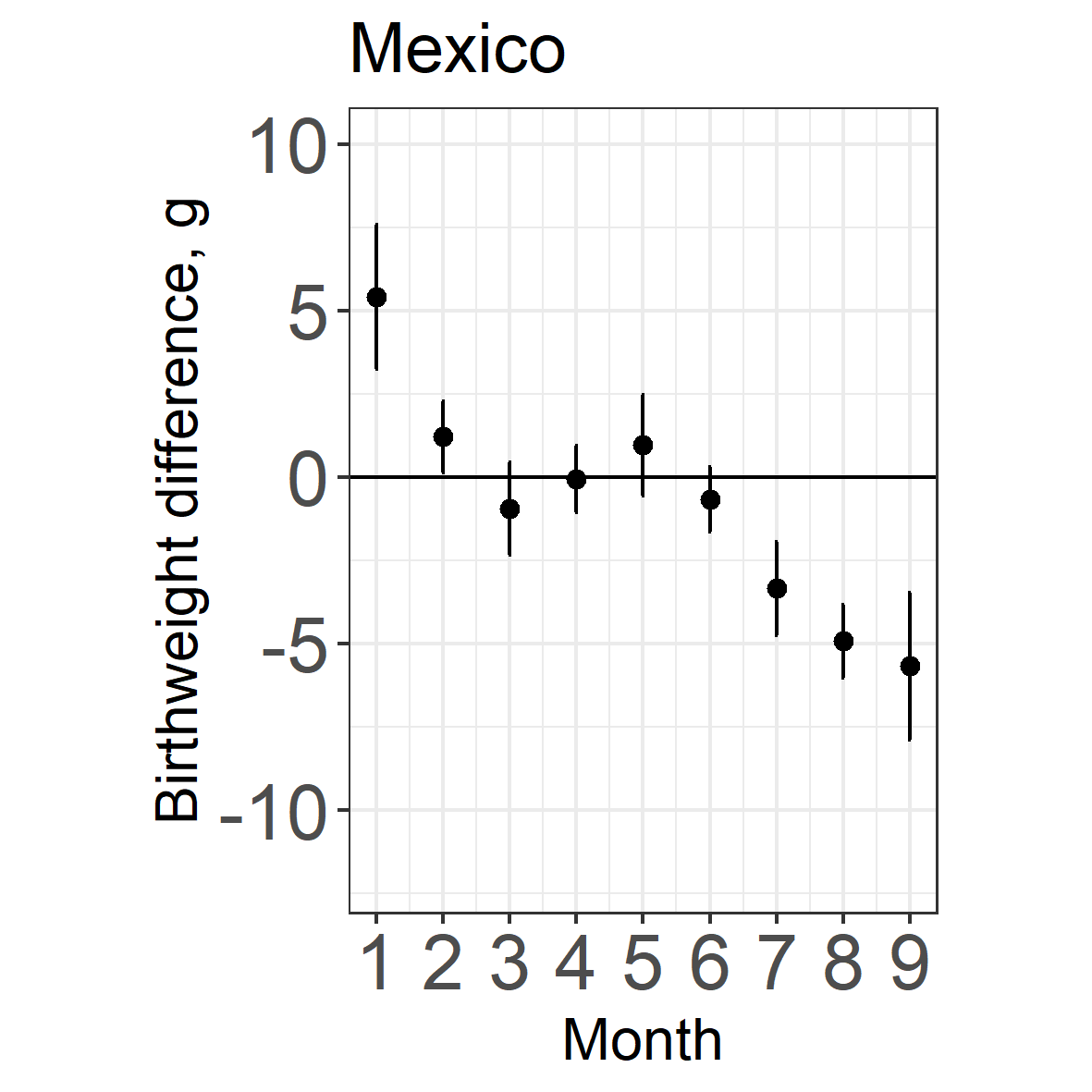

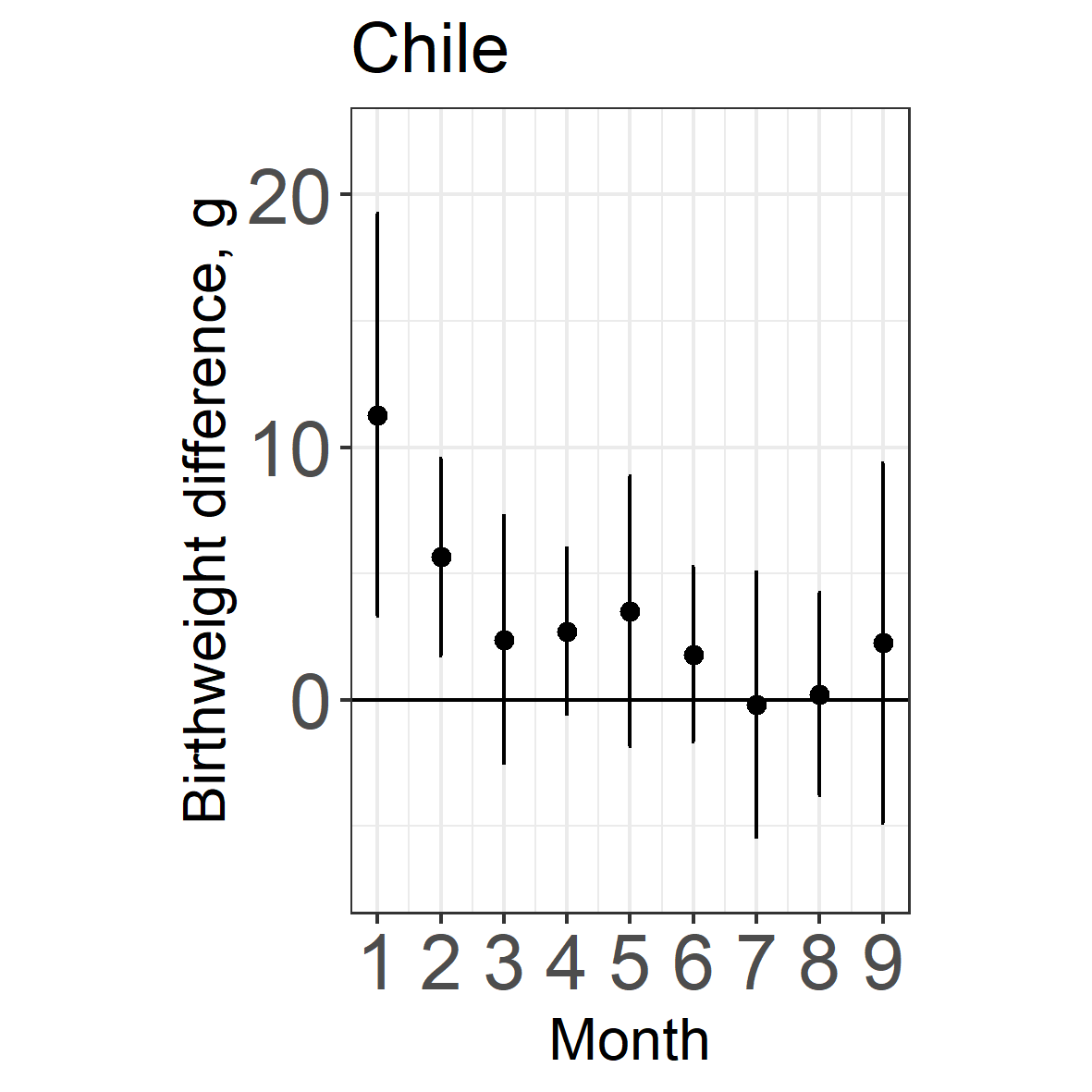


Figure S10. Difference in mean birthweight associated with a 5°C higher temperature in each month of gestation relative to country-specific average monthly temperature (t_avg_ in Brazil = 22.2°C, Mexico = 18.9°C, Chile = 14°C) among term newborns in 2010-2015. The estimates are obtained from the distributed lag non-linear models, adjusted for child sex, mother’s age, education, partnership status, whether the mother had previous births, calendar year of child’s birth, season of conception, climate zone, and include a random intercept for the sub-city of mother’s residence at the time of the child’s birth. Estimates for every exposure window account for temperature exposure during all the other exposure windows during the gestational period.


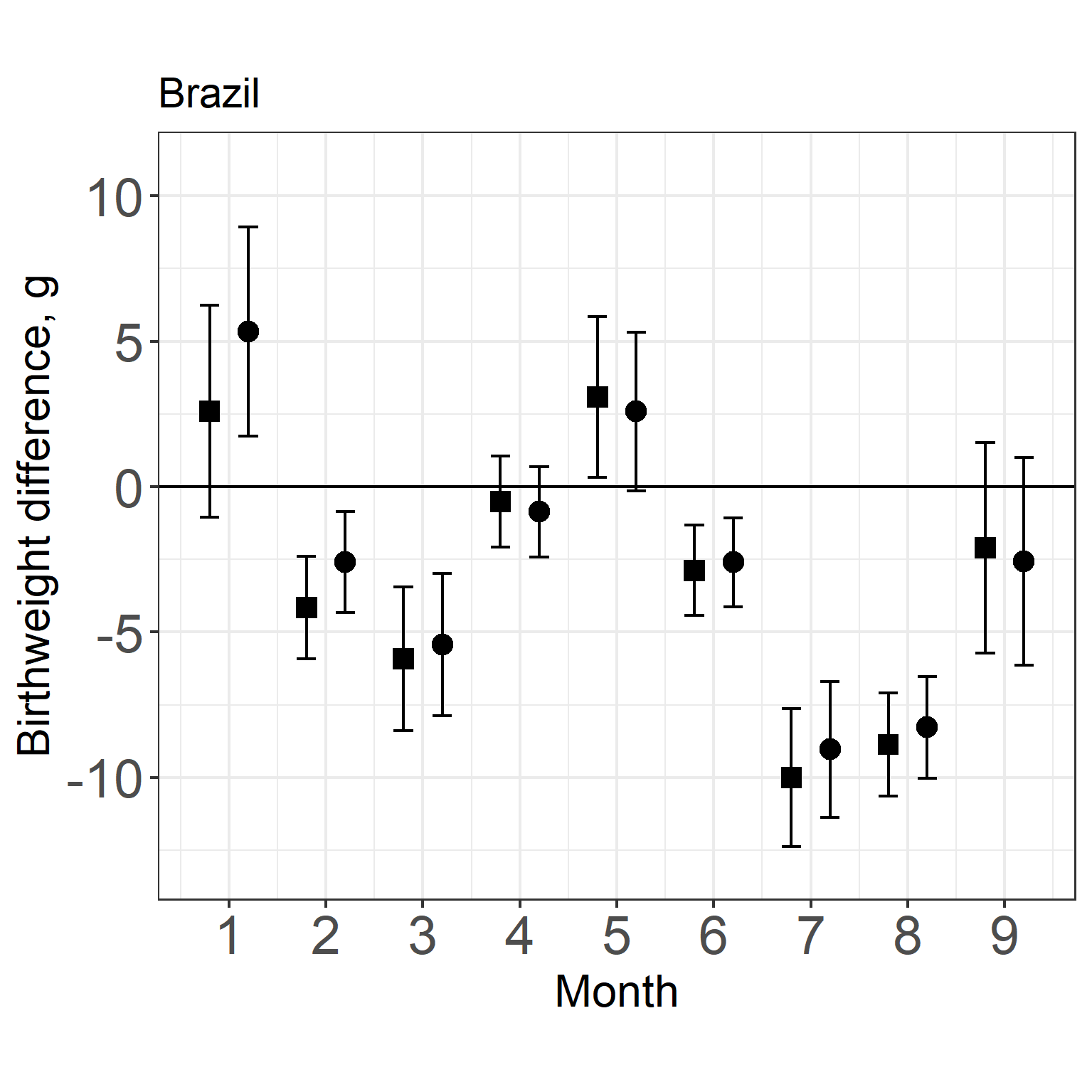


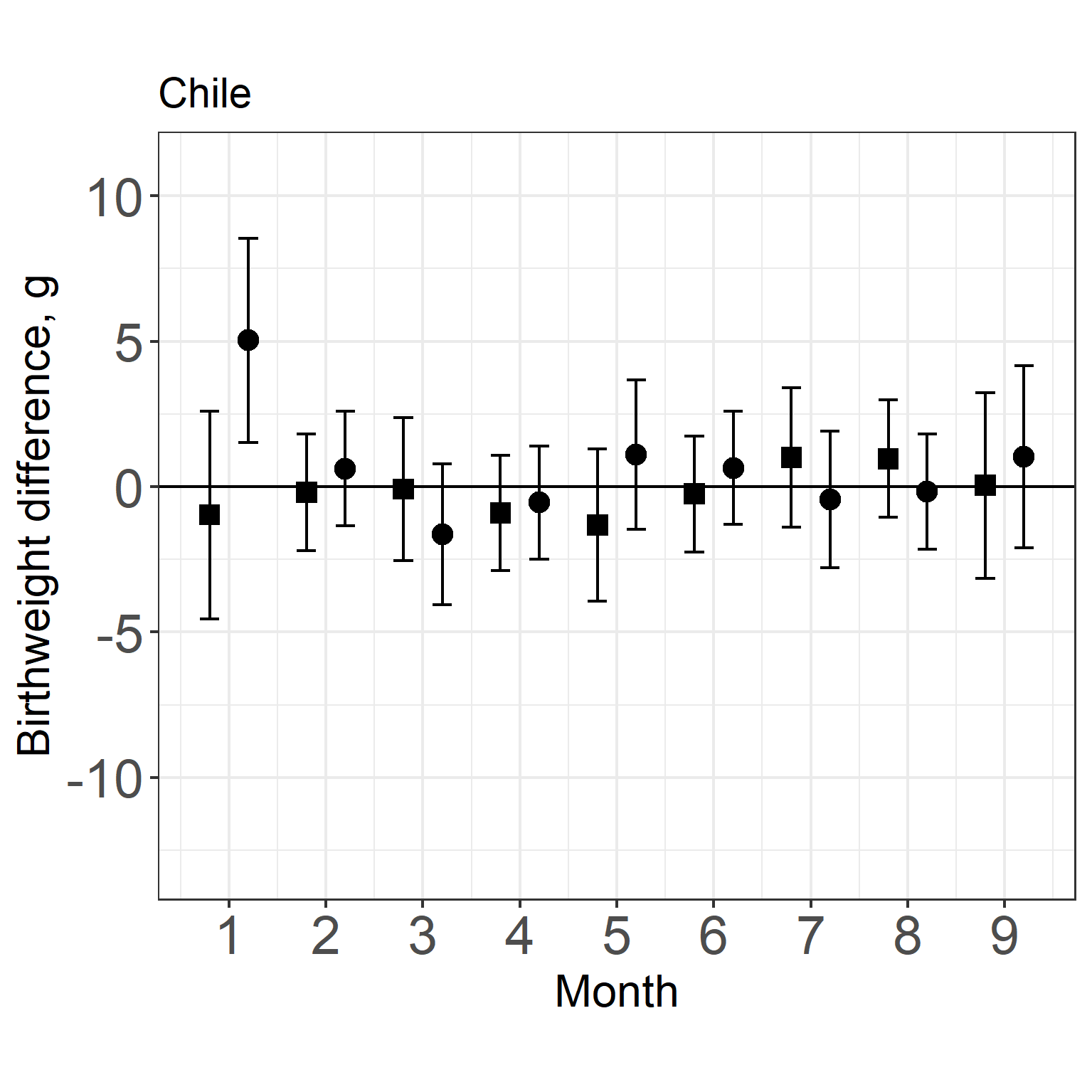

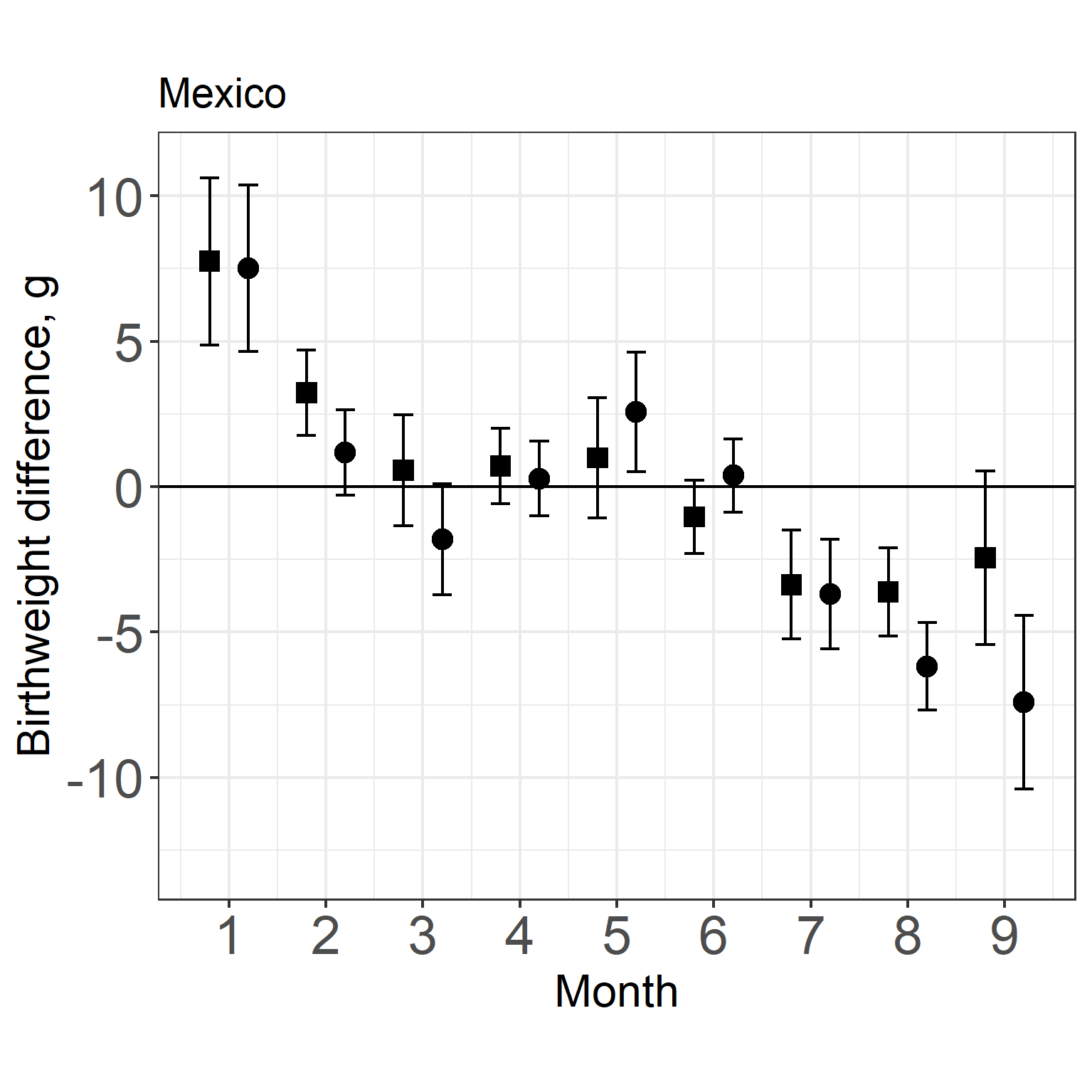

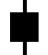

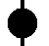


Male

Female

Figure S11. Difference in birthweight associated with a 5°C higher temperature in each month of gestation, relative to a 19°C gestation average (average across the countries) for male and female term newborns in 2010-2015. The estimates are obtained from distributed lag non-linear models stratified by newborn’s sex, adjusted for mother’s age, education, partnership status, whether the mother had previous births, calendar year of child’s birth, season of conception, climate zone, and include a random intercept for the sub-city of mother’s residence at the time of the child’s birth. Estimates for every exposure window account for temperature exposure during all the other exposure windows during the gestational period.

RESULTS OF THE SENSITIVITY ANALYSES

Sensitivity analyses

We performed a number of sensitivity analyses to investigate the robustness of the main results. First, we repeated the main analysis on a sample of live births from nulliparous mothers as nulliparity has been found to be associated with smaller weight and birth and a higher risk of low birthweight.^4^ The results for the nulliparous mothers are in line with the primary analysis (Figure S12).

Second, for Mexico and Chile we re-estimated the models with exposure computed based on the continuous variables of gestational age (recall, the continuous variable was not available for Brazil so to be consistent we relied on the categorical gestational age for the main analyses) (Figure S13). Term births according to the continuous gestational age were selected as those at 38, 39, 40, and 41 weeks of gestation. These results are in line with the primary analysis in the main text.

Third, to check the sensitivity of results in the main text to the measurement of exposure, which is estimated for live births at 38-41 weeks of gestation based on 40-week exposure periods, we estimated models for live births at 37-42 weeks of gestation (Figures S14-S15). We could only do so for Mexico and Chile and not Brazil, as the former two countries have a continuous measure of gestational age in weeks. Because the distributed lag nonlinear models require all observations to have the same lag period, we estimated the models using 42 weeks (or ten months) as the main exposure period. For those births occurring before 42 weeks, we imputed exposure in the missing weeks using temperature from the last observed week.

Fourth, we computed average temperature for every month during the three-month preconception period, in addition to average temperature for the nine months of gestation, and re-estimated the models. These results (Figure S16) did not identify temperature during the preconception as critical, and the results for months 1-9 are in line with those in the main analysis.

Finally, the sub-cities in our sample are characterized by diverse climates and temperature patterns, which could determine biological susceptibility to non-optimal temperatures. We undertook two analyses to investigate the associations between temperature and term birthweight depending on the underlying climatic conditions in an area. First, we repeated the analyses by stratifying the live births by climate zones (Figures S17-S21). The second analysis more closely focused on intra-annual temperature variation since some cities experience little variation in daily temperature throughout the year, whereas some experience substantial fluctuation. For example, Fortaleza, a city in Brazil, has annual daily mean temperature of + 26.7 °C, which varies by an average range of ± 4 °C within a year. To analyze how areas with different patterns of inter-annual temperature variability compare in their temperature-birthweight association, we re-estimated the main models stratified by live births from two categories of sub-cities: 1) sub-cities with an average annual temperature variation ≤ 10°C, as determined by the average annual temperature range during 1996-2015; 2) sub-cities with average annual temperature range > 10°C. Temperature range was computed as the average annual difference between maximum and minimum daily temperature in 1996-2015. (We computed difference between daily max and min temperature for every year in the 1996-2015 period, then averaged those year-specific temperature ranges). There were 107 (out of 422) sub-cities with a limited temperature range in Brazil, 18 (out of 406) in Mexico, and 3 in Chile (out of 81) (Figure S22). These results (Figure S23) are in line with the main text, except for the low-range sub-cities in Brazil, where we observe negative associations between higher temperatures and birthweight in the first several months of gestation, as opposed to the last months of gestation from the primary analysis.

1. Live births from nulliparous women


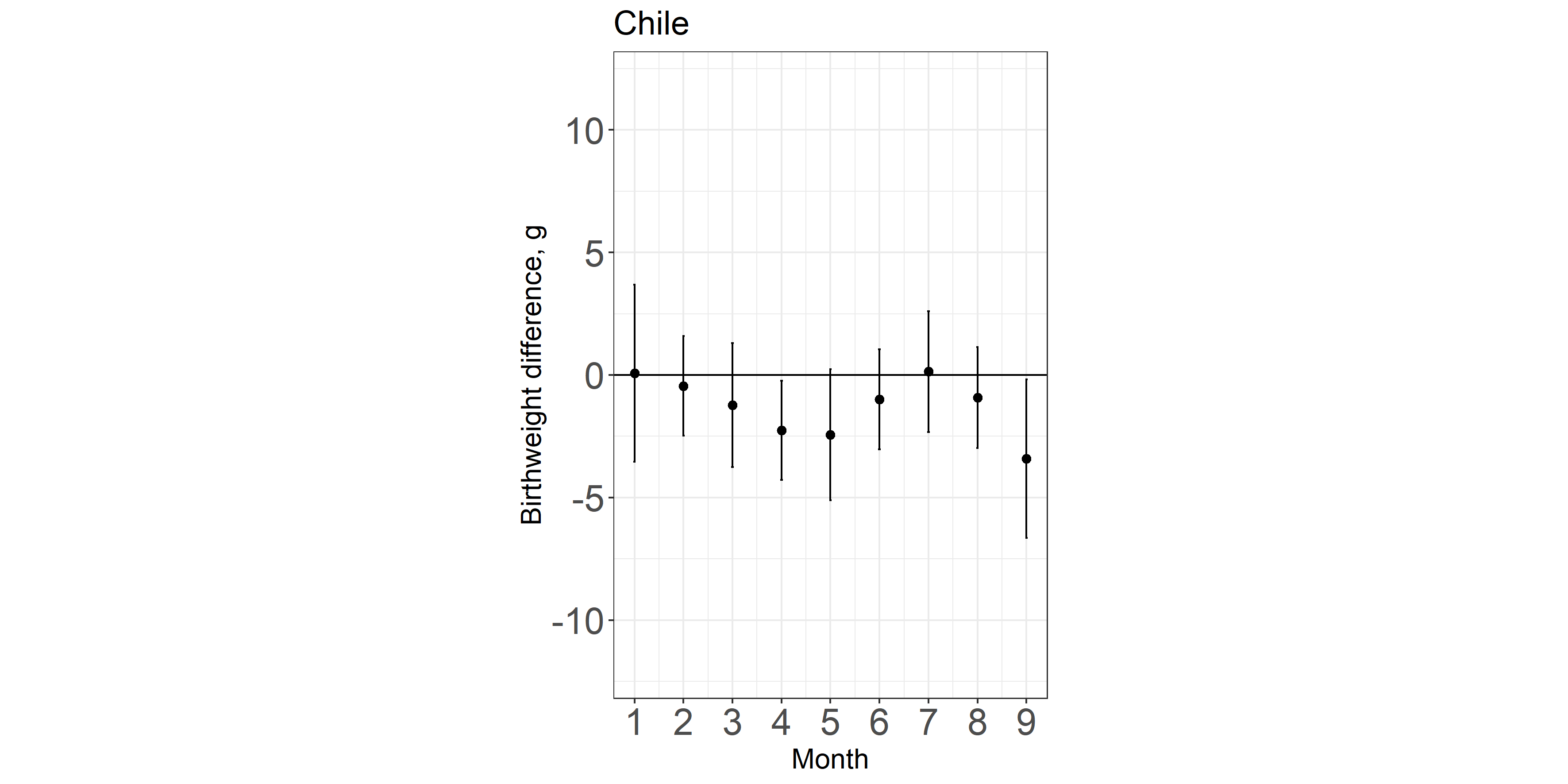

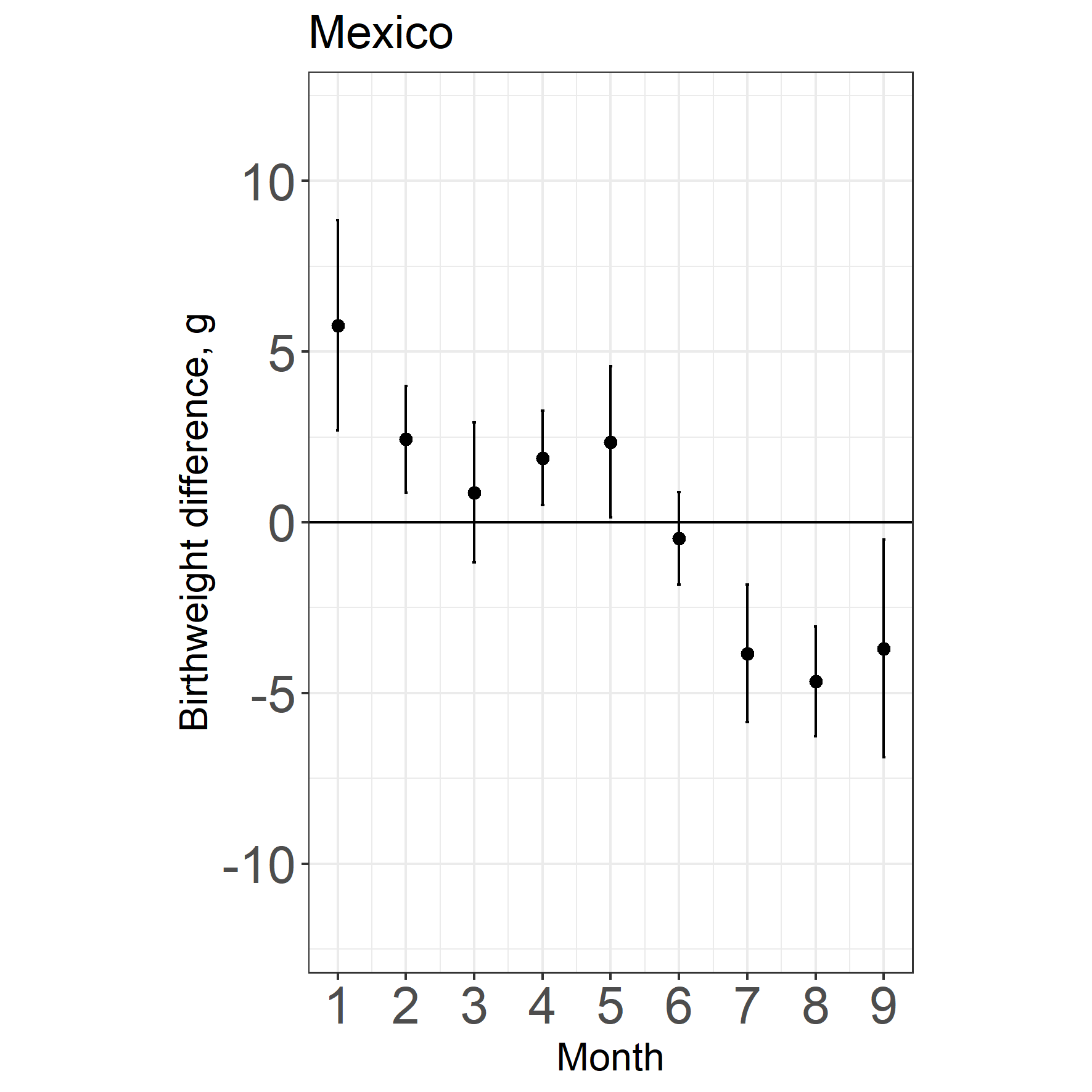

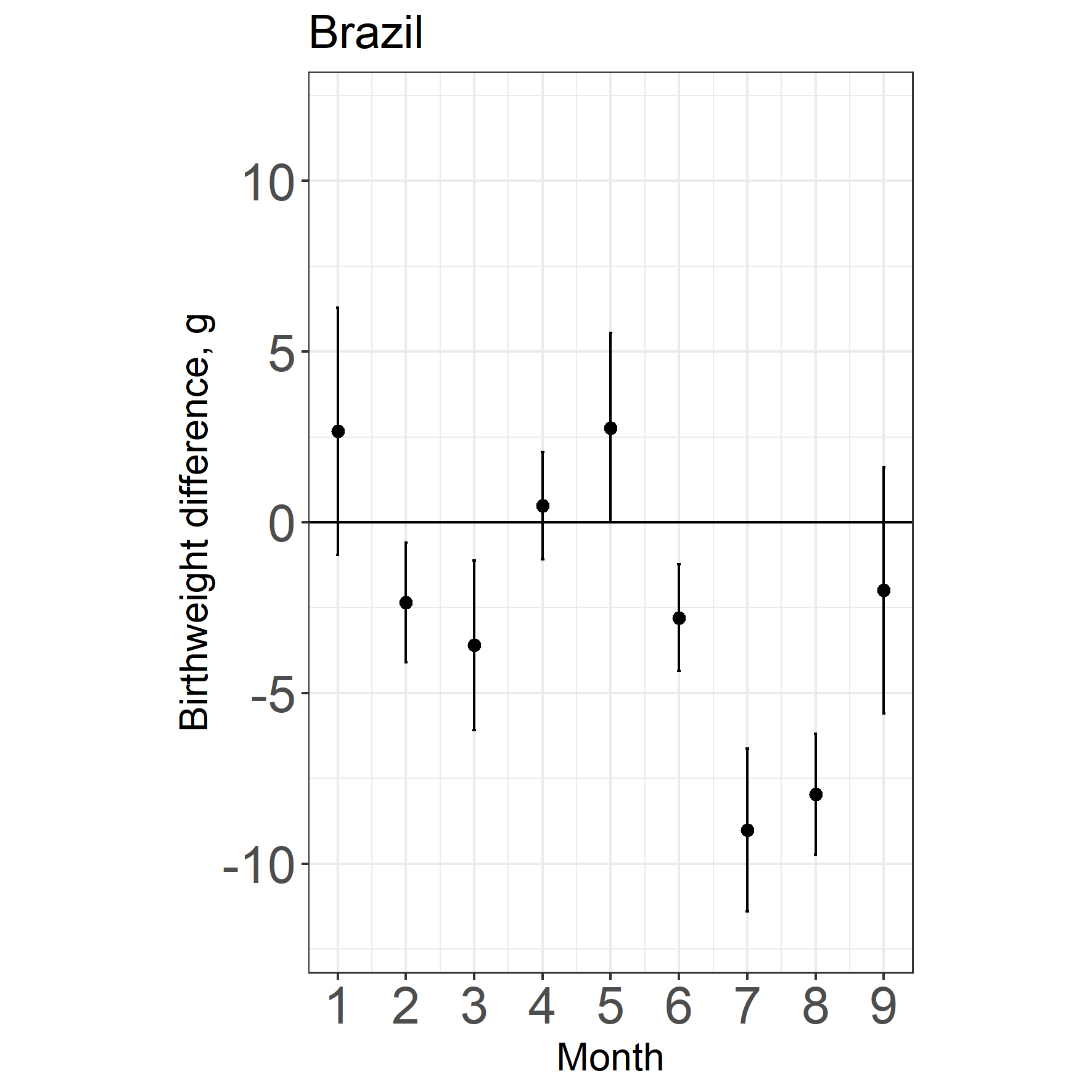


Figure S12. Difference in mean birthweight associated with a 5°C higher temperature in each month of gestation relative to a 19°C gestation average (average across the countries) among term newborns in 2010-2015, for nulliparous women. The estimates are obtained from distributed lag non-linear models, adjusted for child sex, mother’s age, education, partnership status, calendar year of child’s birth, climate zone, season of conception, and include a random intercept for the sub-city of mother’s residence at the time of the child’s birth. Estimates for every exposure window account for temperature exposure during all the other exposure windows during the gestational period.

1. Temperature exposure computed using the continuous measure of gestational age in weeks (available for Mexico and Chile).


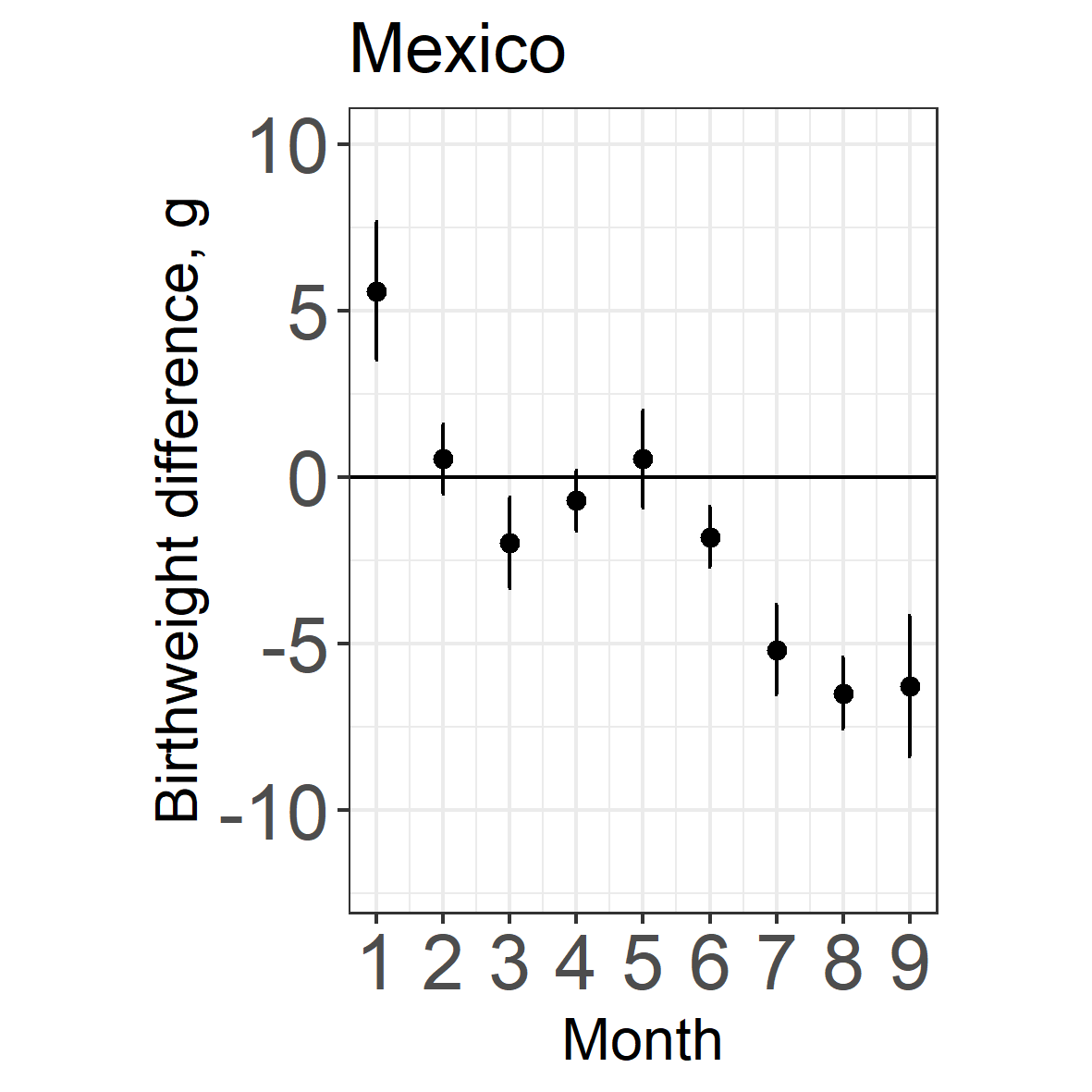

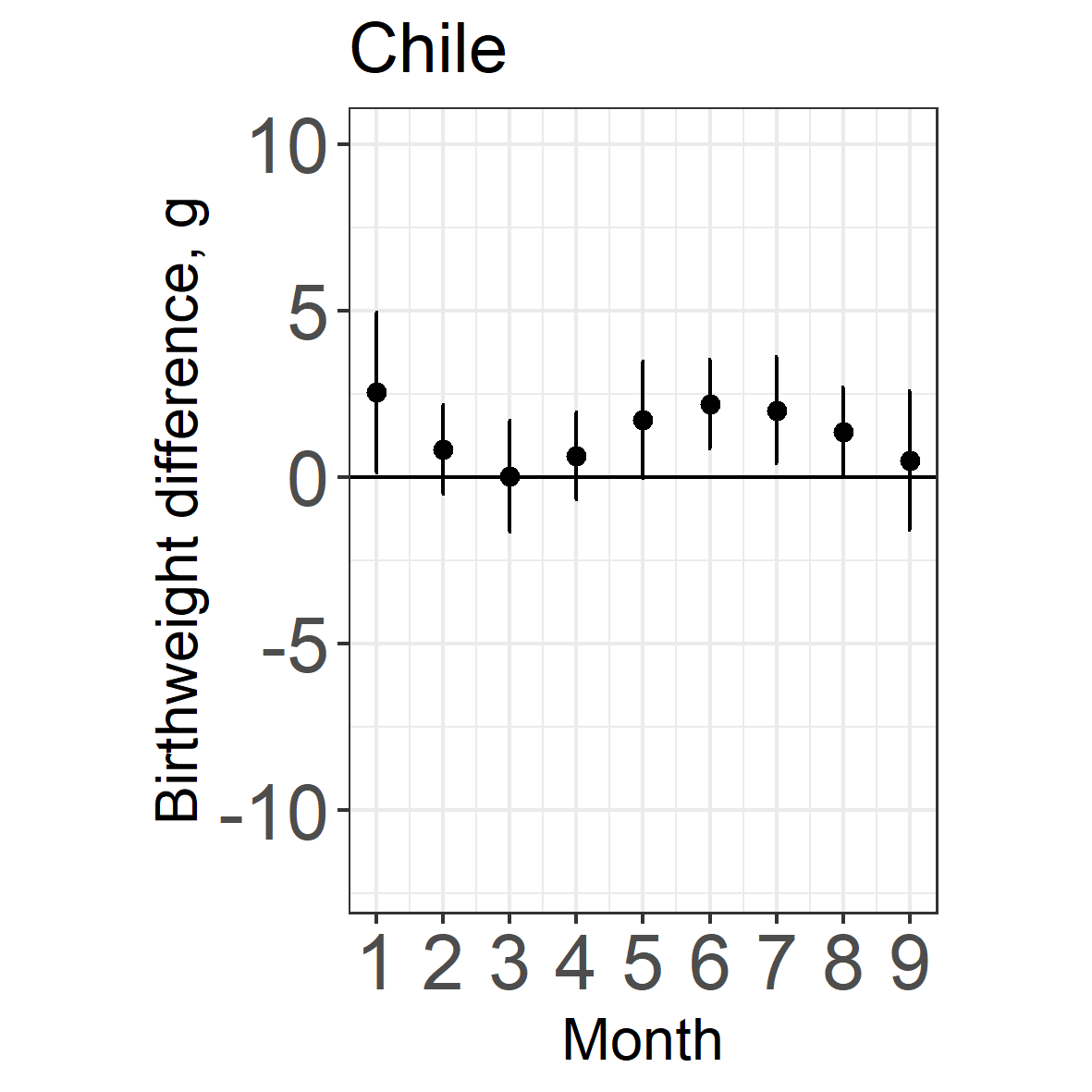


Figure S13. Difference in birthweight associated with a 5°C higher temperature in each month of pregnancy, relative to a 19°C reference, among term newborns in 2010-2015. The estimates are obtained from distributed lag non-linear models, adjusted for child sex, gestational age category (38, 39, 40 weeks), mother’s age, education, partnership status, calendar year of child’s birth, season of conception, climate zone, and include a random intercept for the sub-city of mother’s residence at the time of the child’s birth. Temperature exposure during gestation for these births was computed based on the exact gestational age in weeks (38, 39, or 40 weeks). Estimates for every exposure window account for temperature exposure during all the other exposure windows during the gestational period.

1. Temperature exposure computed using the continuous measure of gestational age in weeks (available for Mexico and Chile) for newborns born between 37-42 weeks of gestation.


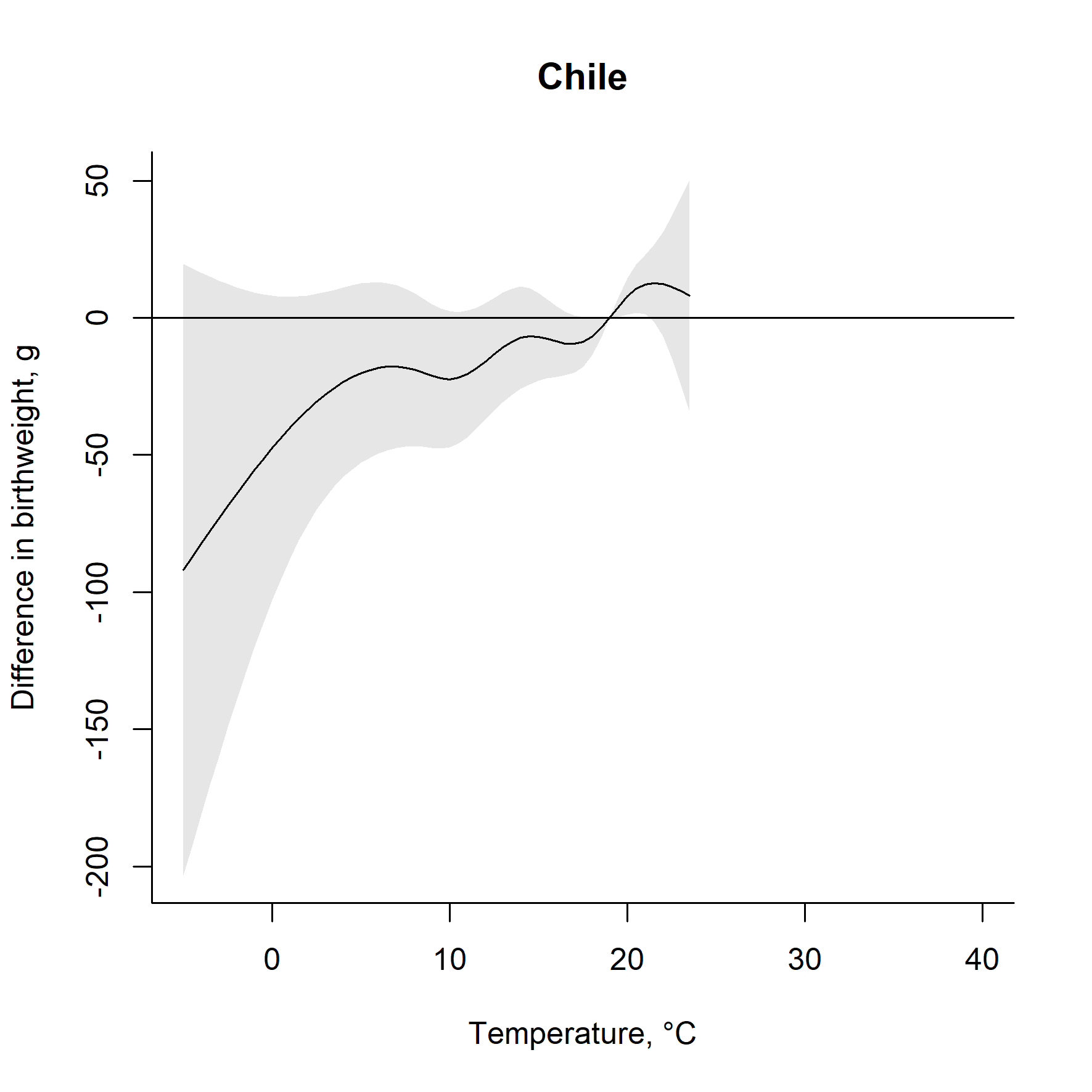

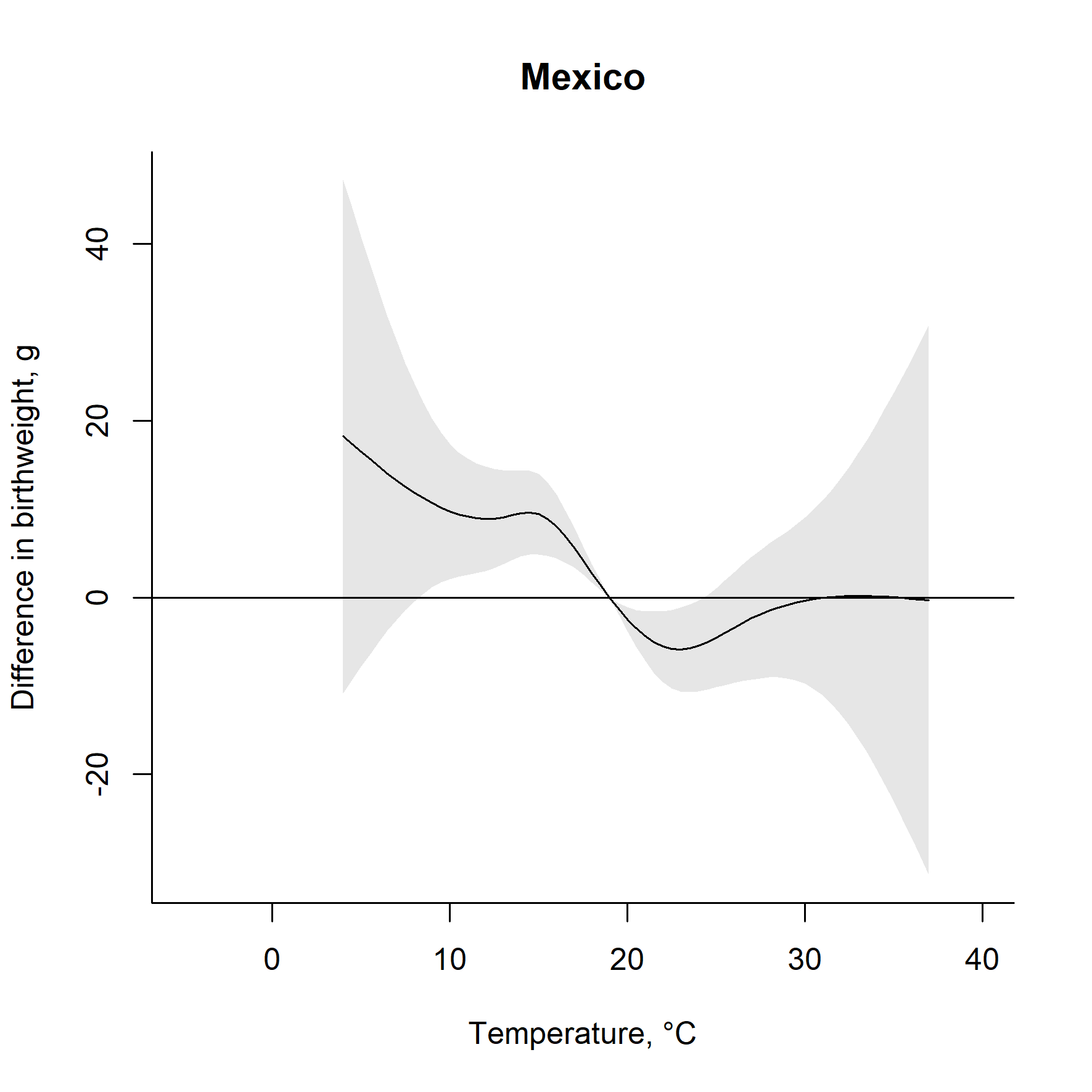


Figure S14. Cumulative associations between average monthly temperature during the 37-42 week gestational period and birthweight for term newborns during 2010-2015 in Mexico and Chile. The estimates are obtained from distributed lag non-linear models, adjusted for child sex, gestational age, mother’s age, education, partnership status, calendar year of child’s birth, season of conception, climate zone, and include a random intercept for the sub-city of mother’s residence at the time of the child’s birth. Because the distributed lag nonlinear models require all observations to have the same lag period, we estimated the models using 42 weeks (or ten months) as the main exposure period. For those births occurring before 42 weeks, we imputed exposure in the missing weeks using temperature from the last observed week. Temperature on the x-axis refers to the average monthly temperature during the entire gestation.


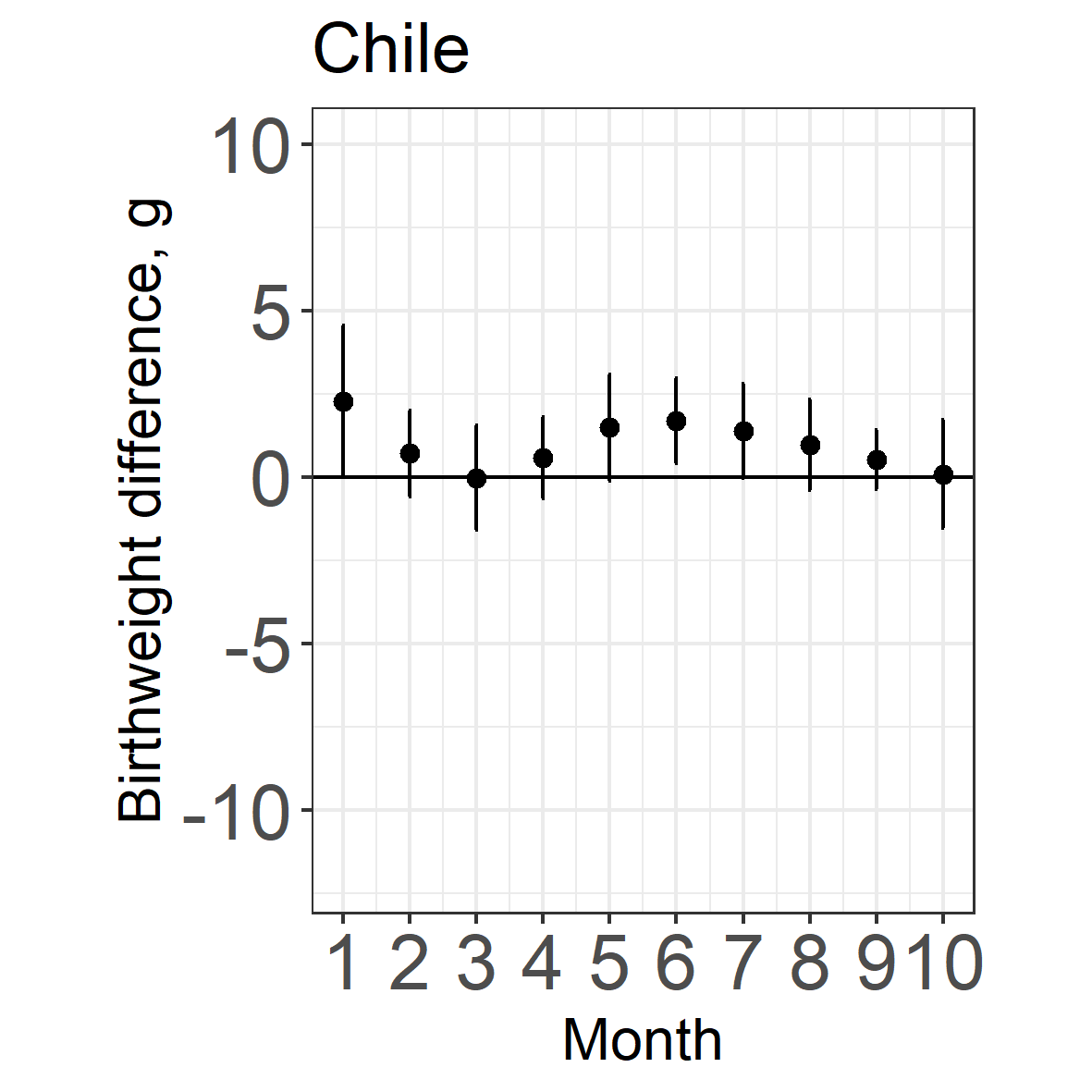

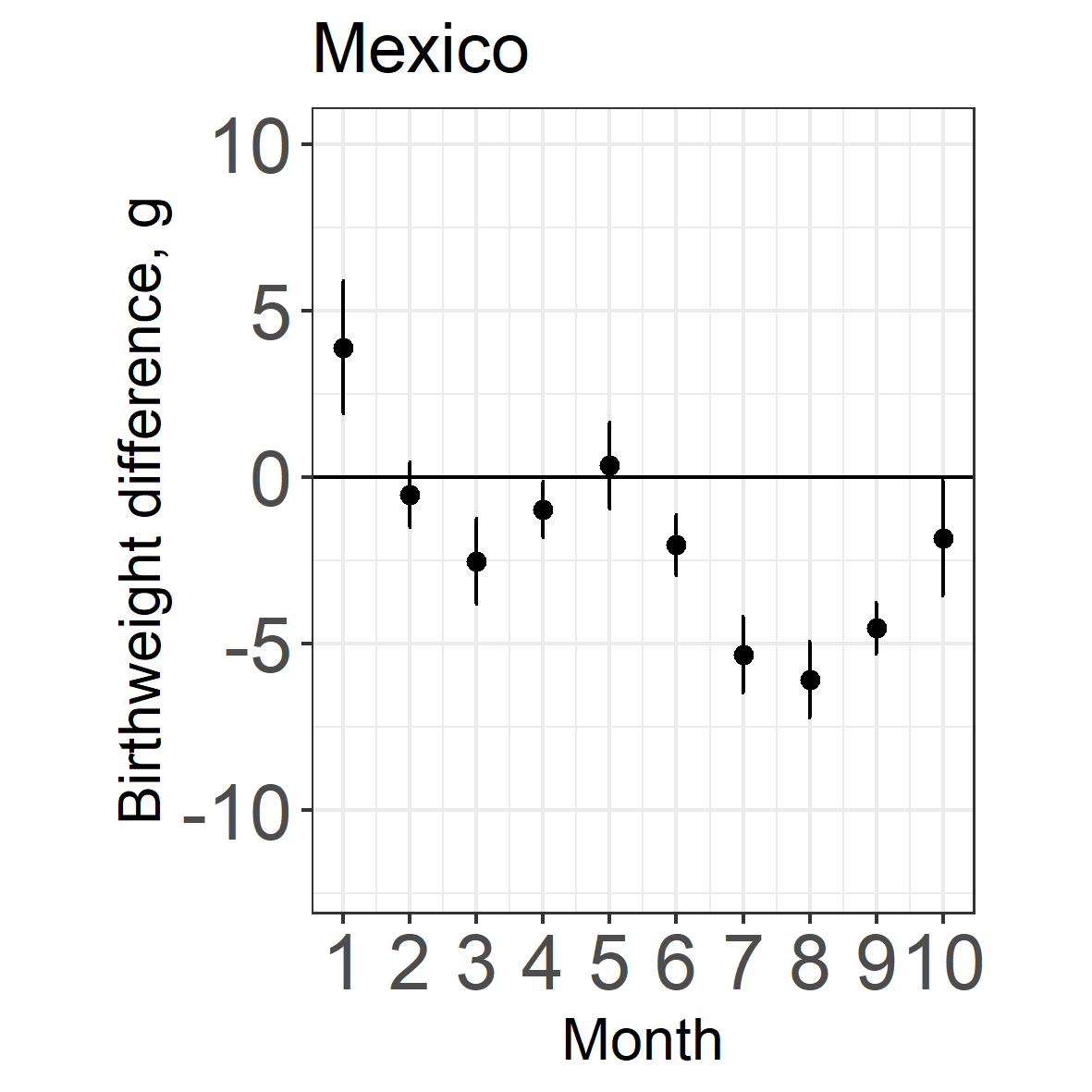


Figure S15. Difference in birthweight associated with a 5°C higher temperature in each month of pregnancy, relative to a 19°C reference, among live newborns born at 37-42 weeks of gestation during 2010-2015 in Mexico and Chile. The estimates are obtained from distributed lag non-linear models, adjusted for child sex, gestational age, mother’s age, education, partnership status, calendar year of child’s birth, season of conception, climate zone, and include a random intercept for the sub-city of mother’s residence at the time of the child’s birth. Because the distributed lag nonlinear models require all observations to have the same lag period, we estimated the models using 42 weeks (or ten months) as the main exposure period. For those births occurring before 42 weeks, we imputed exposure in the missing weeks using temperature from the last observed week. Estimates for every exposure window account for temperature exposure during all the other exposure windows during the gestational period.

1. Temperature exposure during gestation and a three-month preconception period


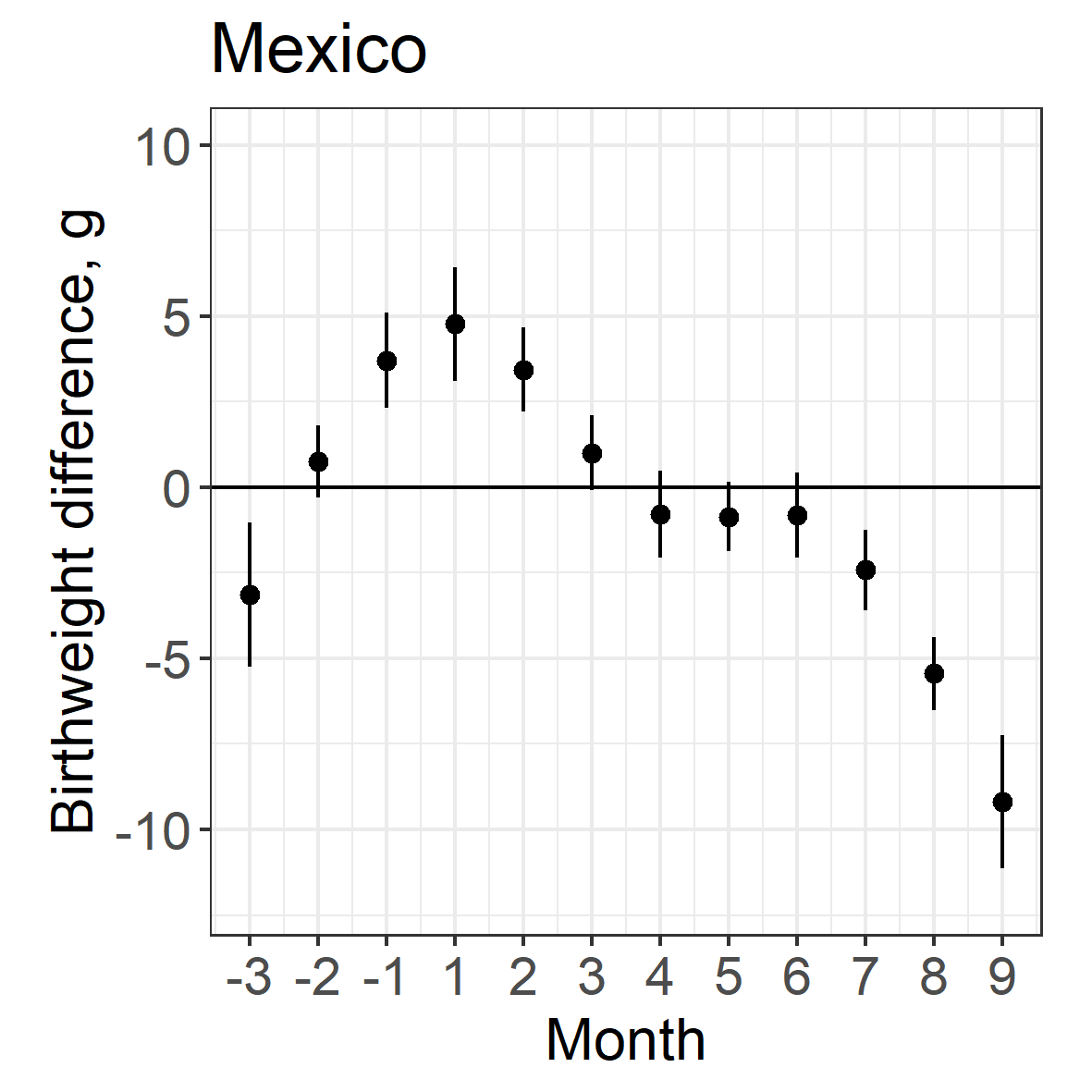

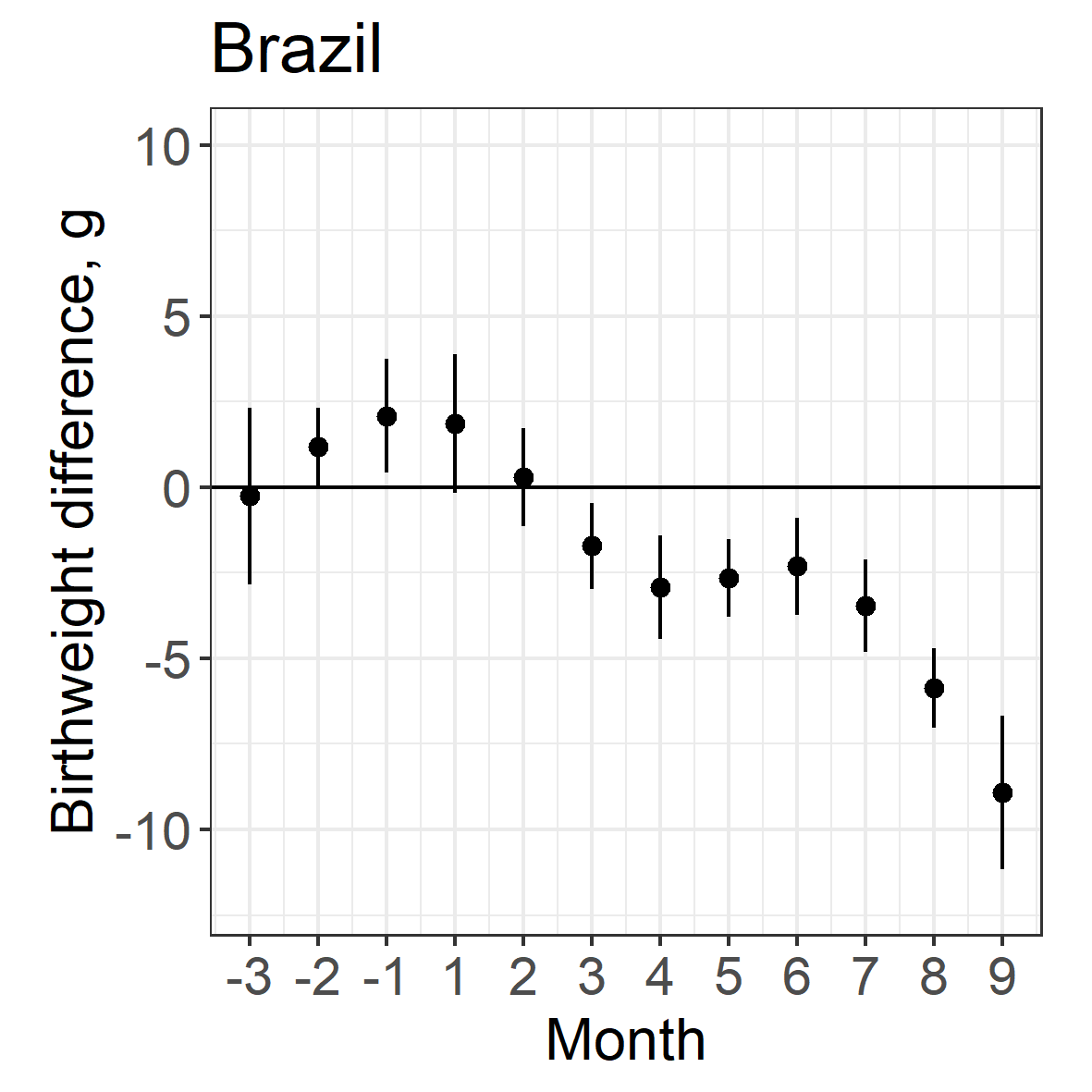

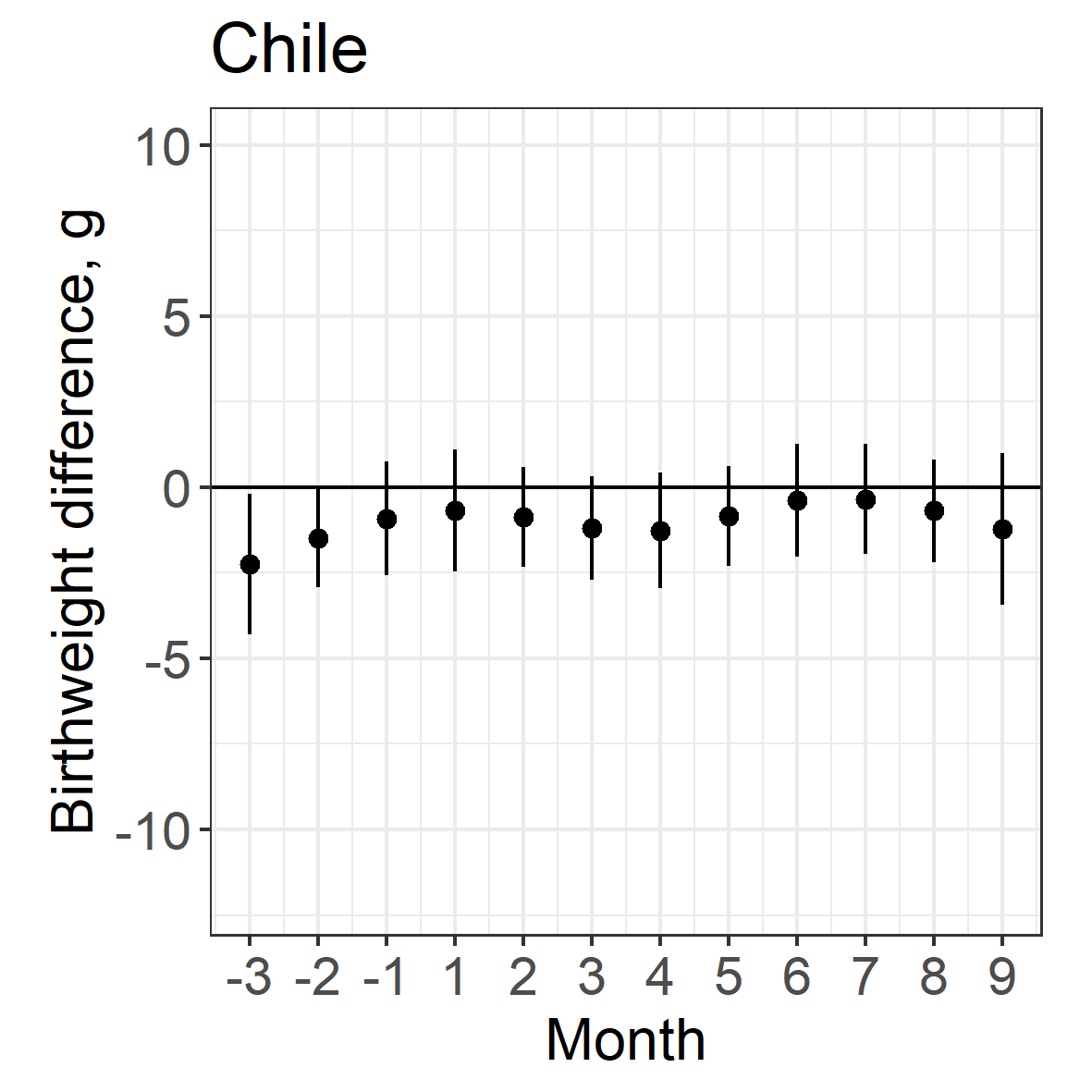


Figure S16. Difference in birthweight associated with a 5°C higher temperature in each month of gestation and three months before conception, relative to a 19°C gestation average reference temperature, among term newborns in 2010-2015. The estimates are obtained from distributed lag non-linear models, adjusted for child sex, mother’s age, education, partnership status, whether the mother had previous births, calendar year of child’s birth, season of conception, climate zone, and include a random intercept for the sub-city of mother’s residence at the time of the child’s birth. Estimates for every exposure window account for temperature exposure during all the other exposure windows during the gestational period.

1. Results stratified by climate zone


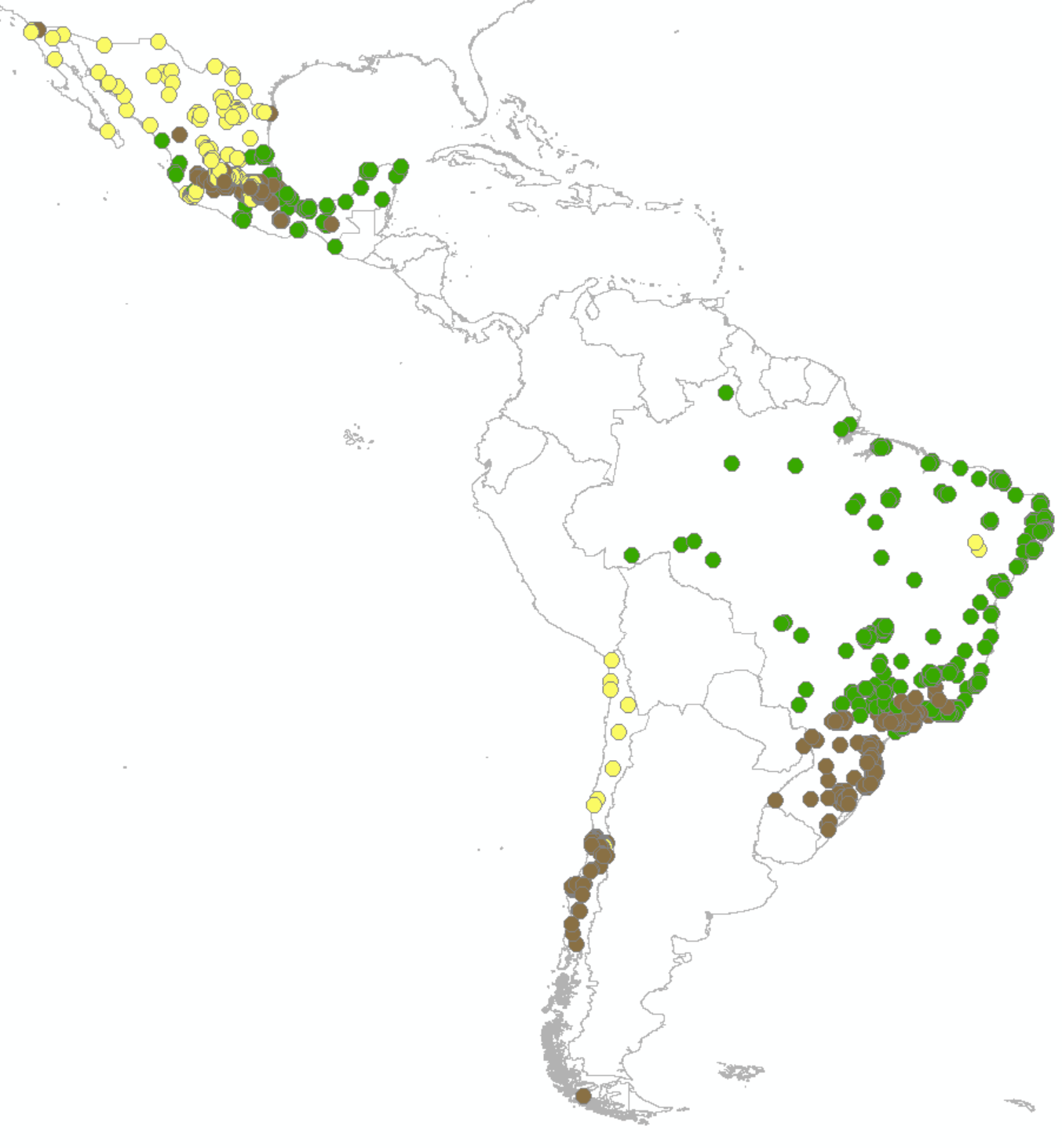

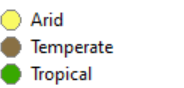


Figure S17. Sub-cities and their climate zones in Brazil, Mexico and Chile


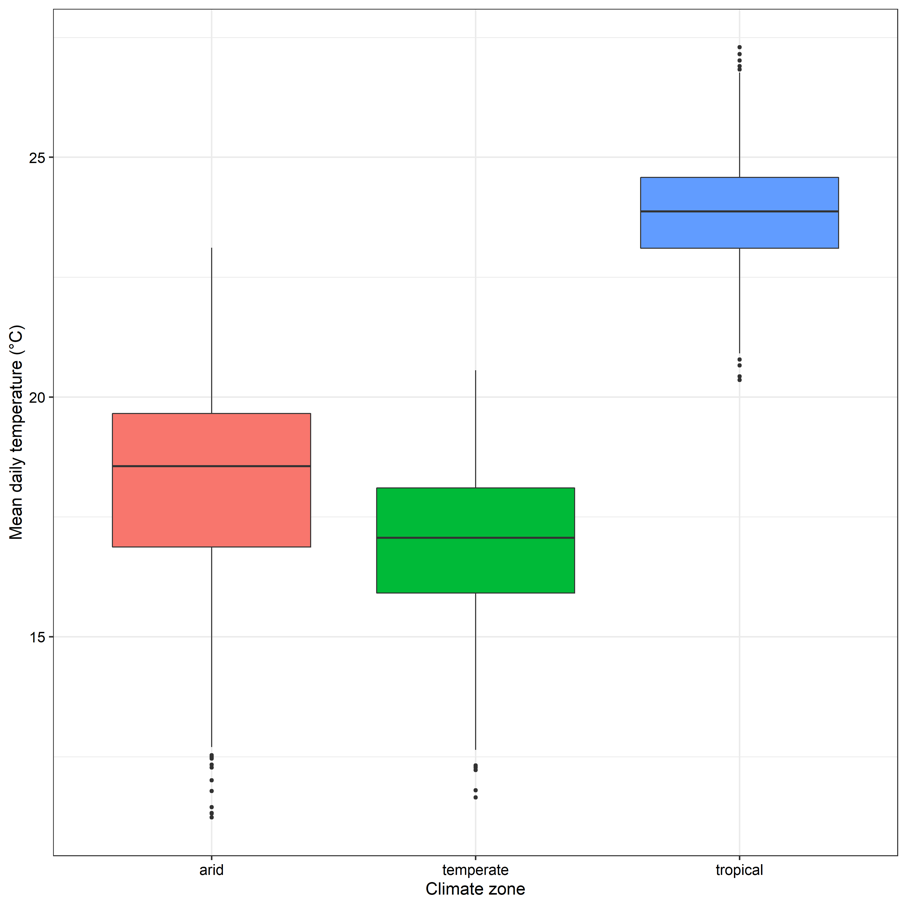


Figure S18. Distributions of mean daily temperature during 2010-2015 in the sub-cities of Brazil, Mexico, and Chile by climate zone.


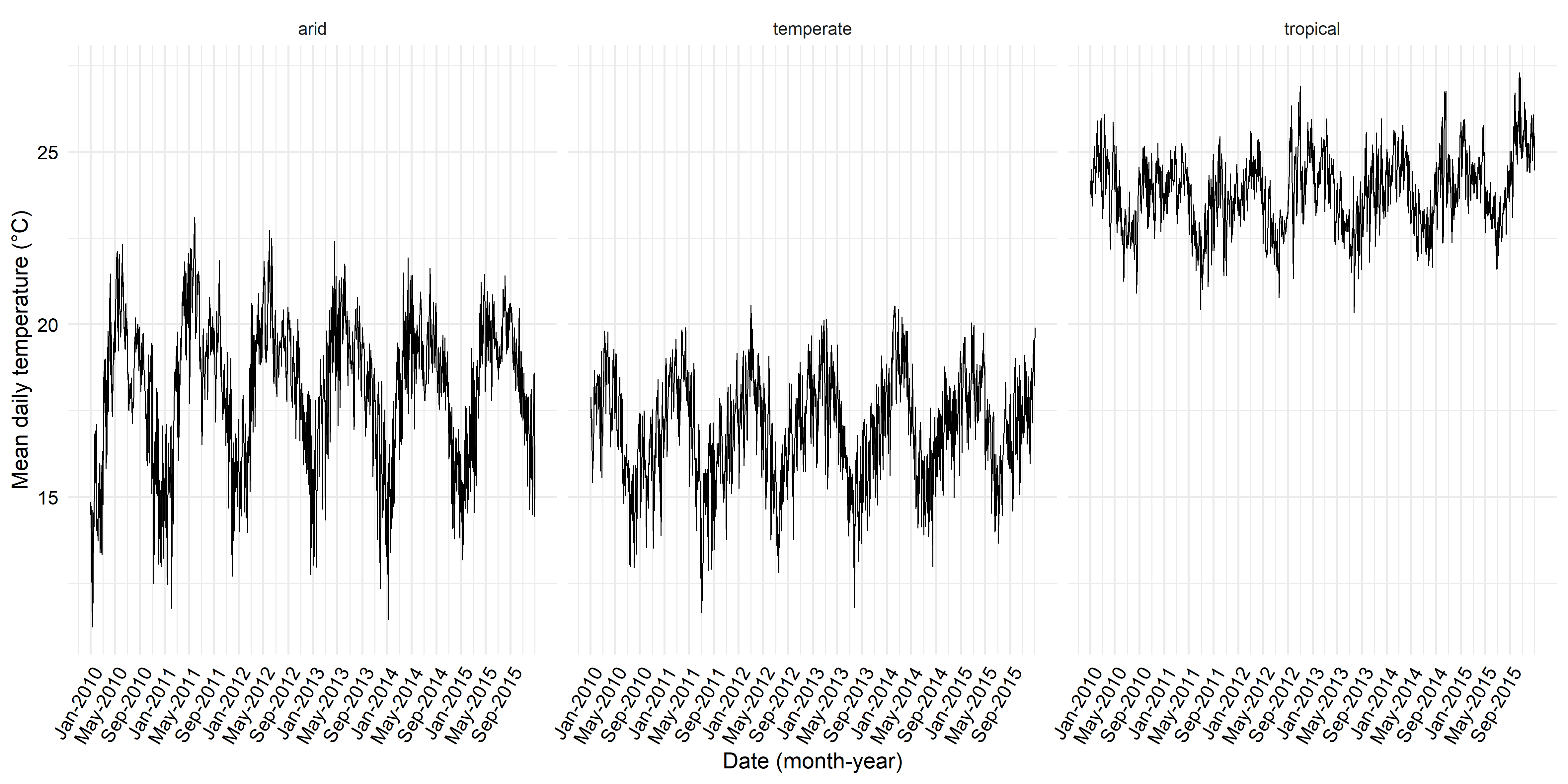


Figure S19. Seasonal variation of mean daily temperature in the sub-cities of Brazil, Mexico, and Chile

by climate zone.


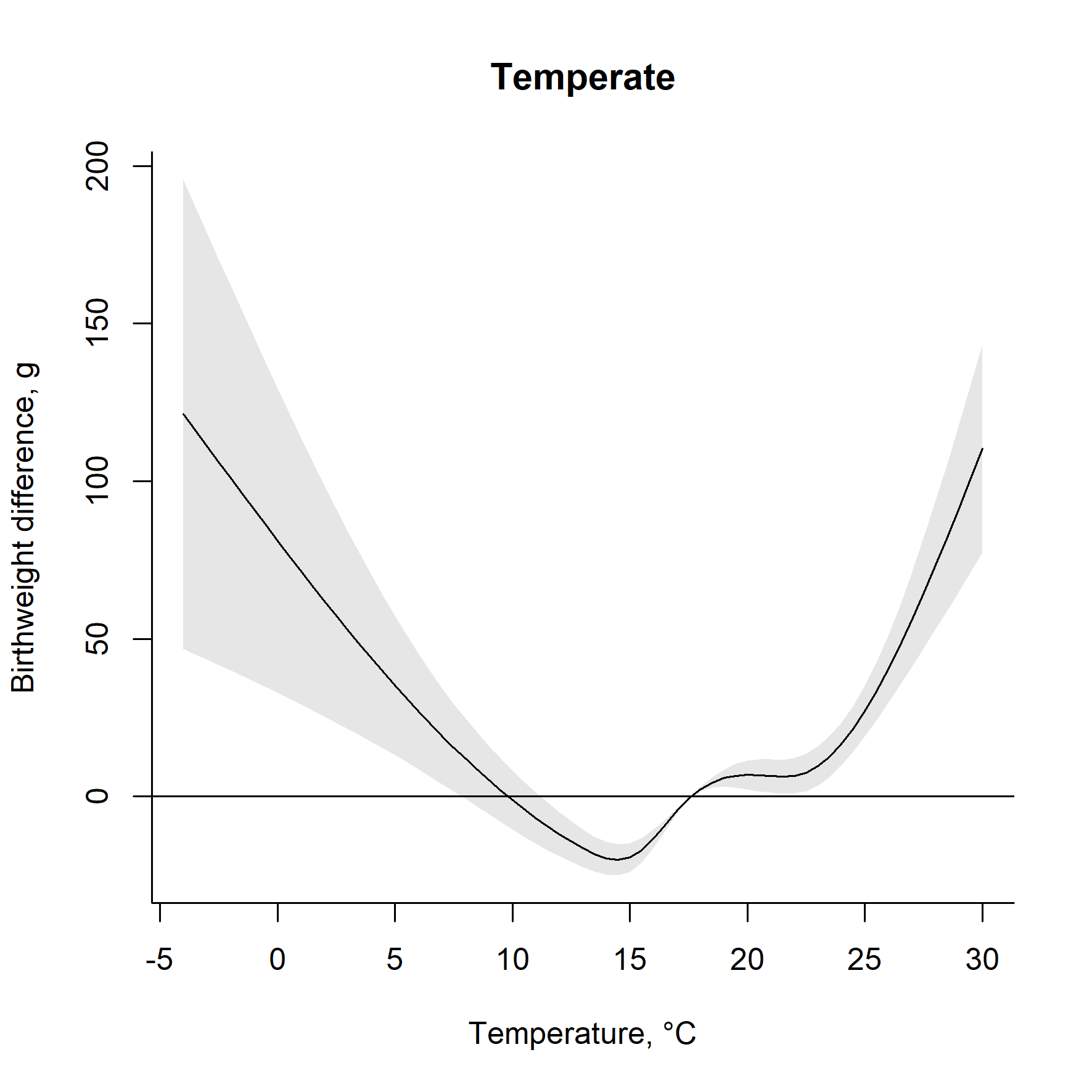

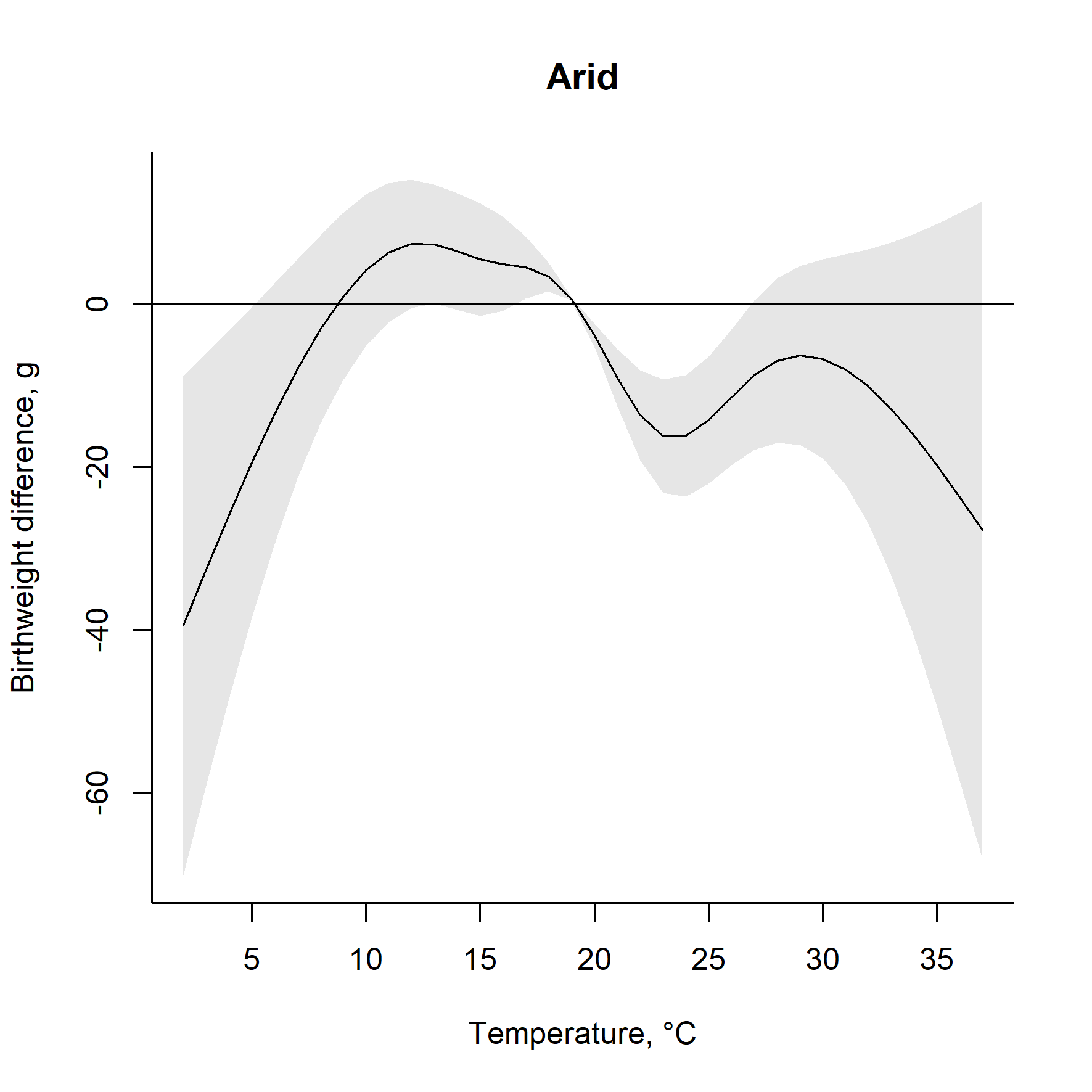

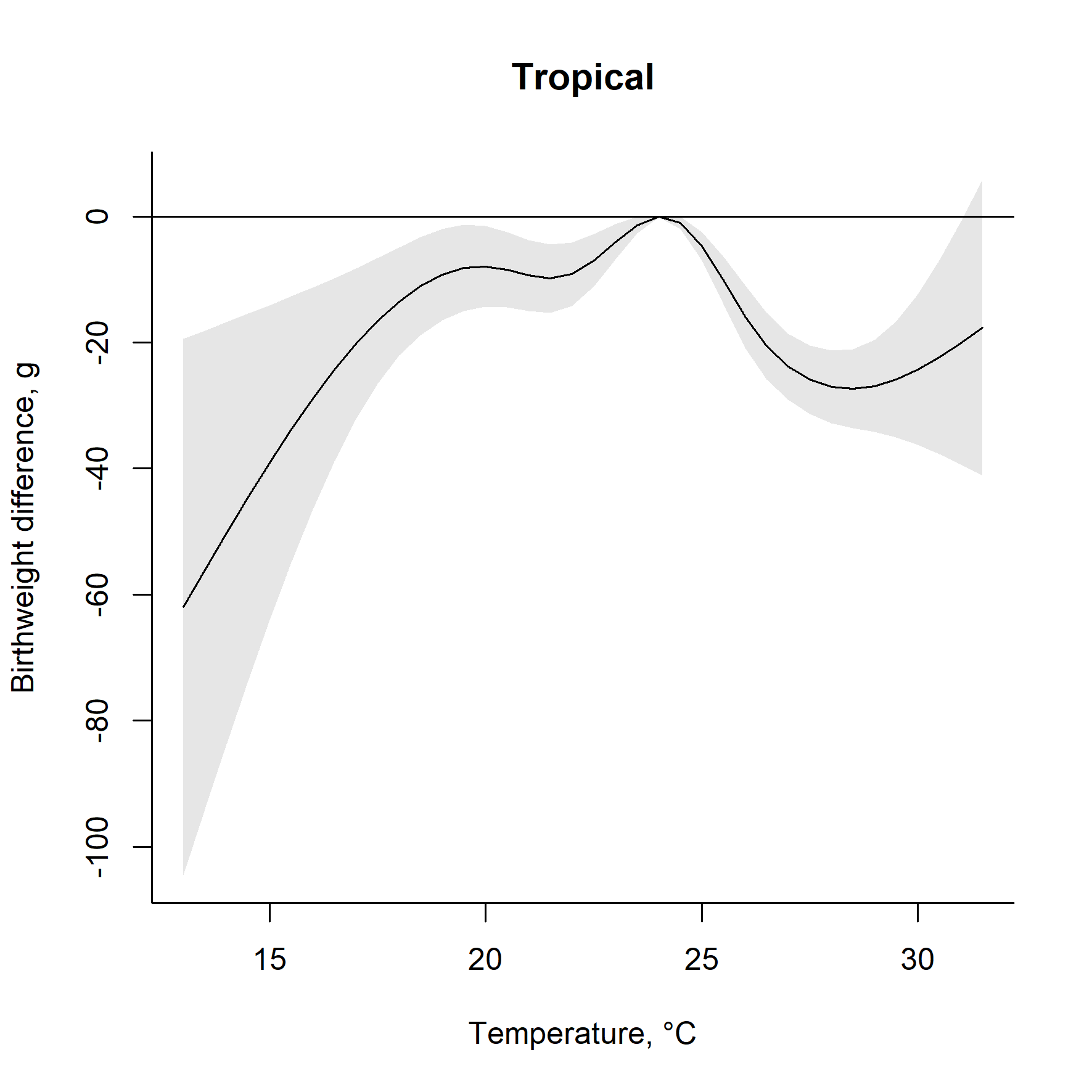


Figure S20. Cumulative associations between average monthly temperature during nine months of gestation and birthweight for term newborns in 2010-2015 by climate zones. The curves depict estimated difference in birthweight associated with average temperature during gestation relative to a reference temperature of 19°C (average monthly temperature across the countries). The curves are derived from distributed lag non-linear models stratified by climate zones and adjusted for child sex, mother’s age, education, partnership status, whether she had previous births, calendar year of child’s birth, season of conception, country fixed effects, and include random intercepts for the sub-city of mother’s residence at the time of the child’s birth. Temperature on the x-axis refers to the average monthly temperature during the entire gestation.


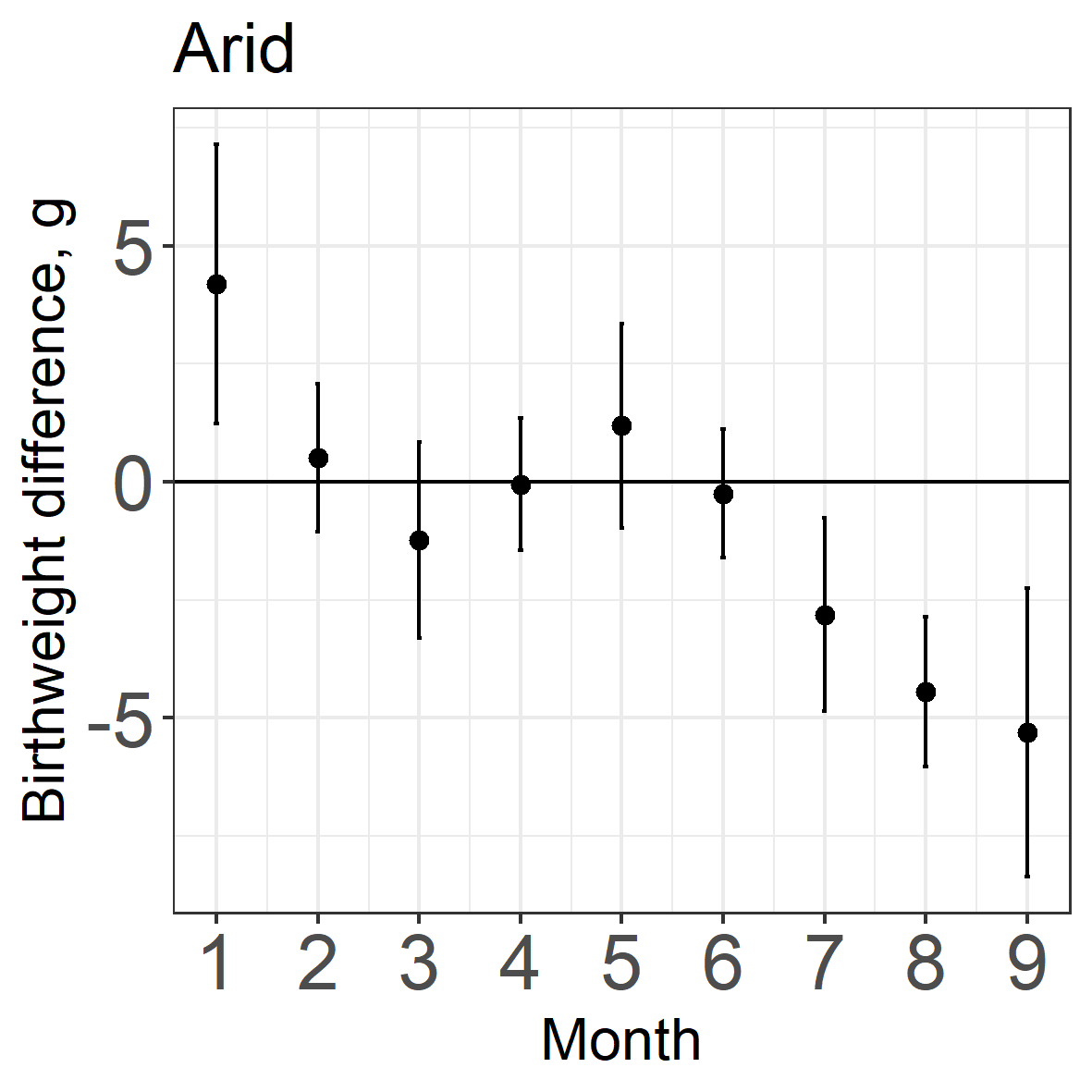

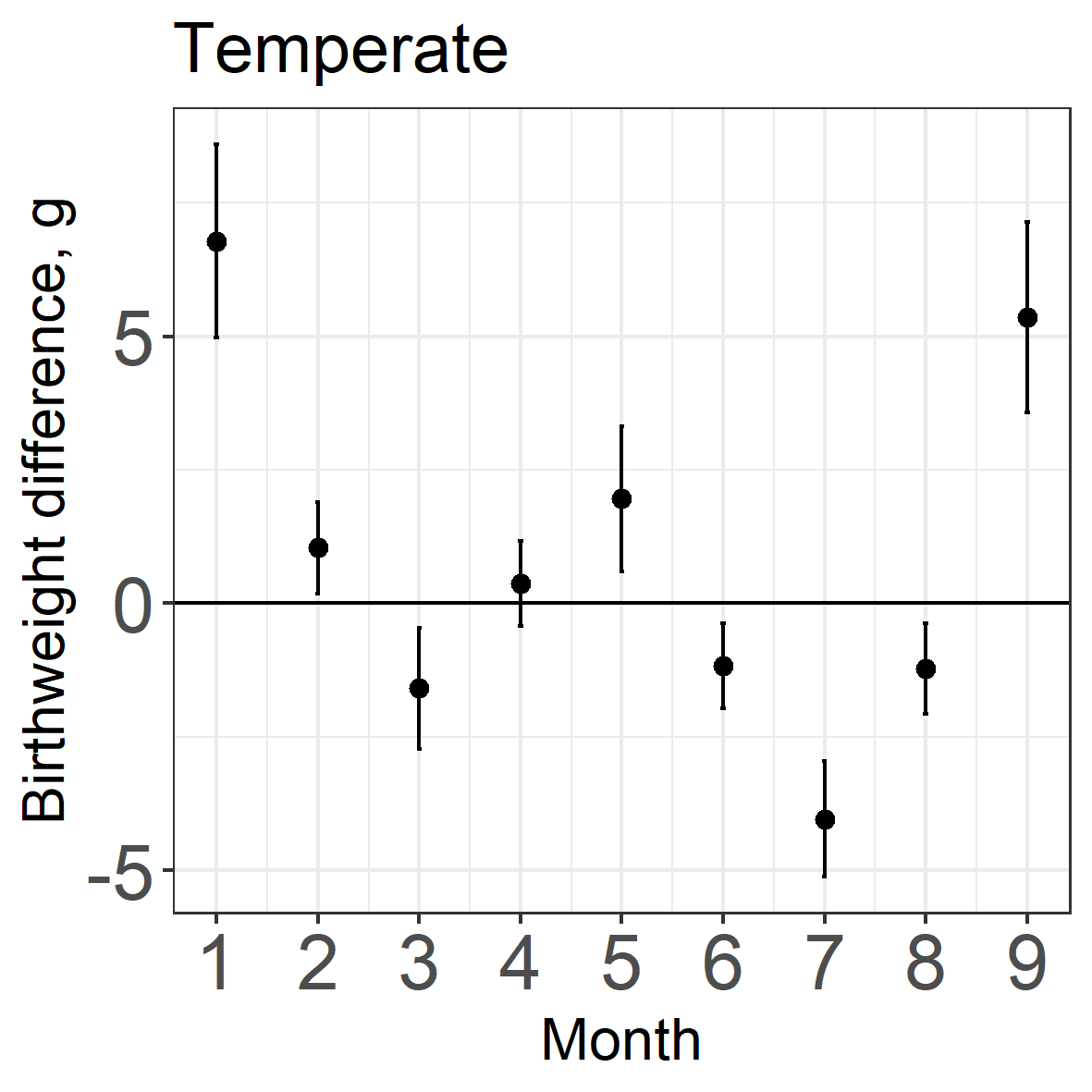

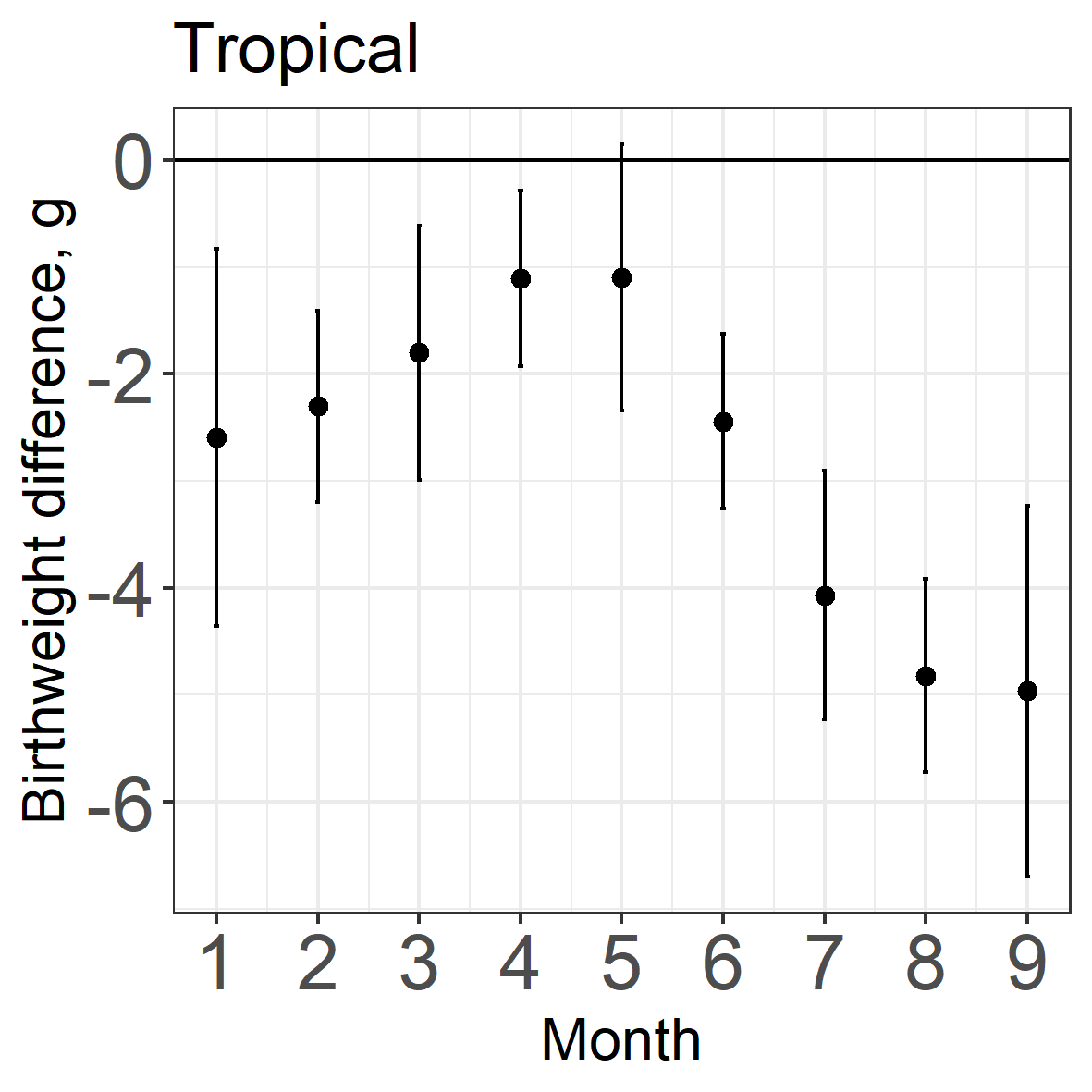


Figure S21. Difference in mean birthweight associated with a 5°C higher temperature in each month of gestation relative to a 19°C gestation average (average across the countries) among term newborns in 2010-2015 by climate zone. The estimates are obtained from distributed lag non-linear models stratified by climate zone and adjusted for child sex, mother’s age, education, partnership status, whether the mother had previous births, calendar year of child’s birth, season of conception, country fixed effects, and include a random intercept for the sub-city of mother’s residence at the time of the child’s birth. Estimates for every exposure window account for temperature exposure during all the other exposure windows during the gestational period.

1. Results from sub-cities with different temperature variation


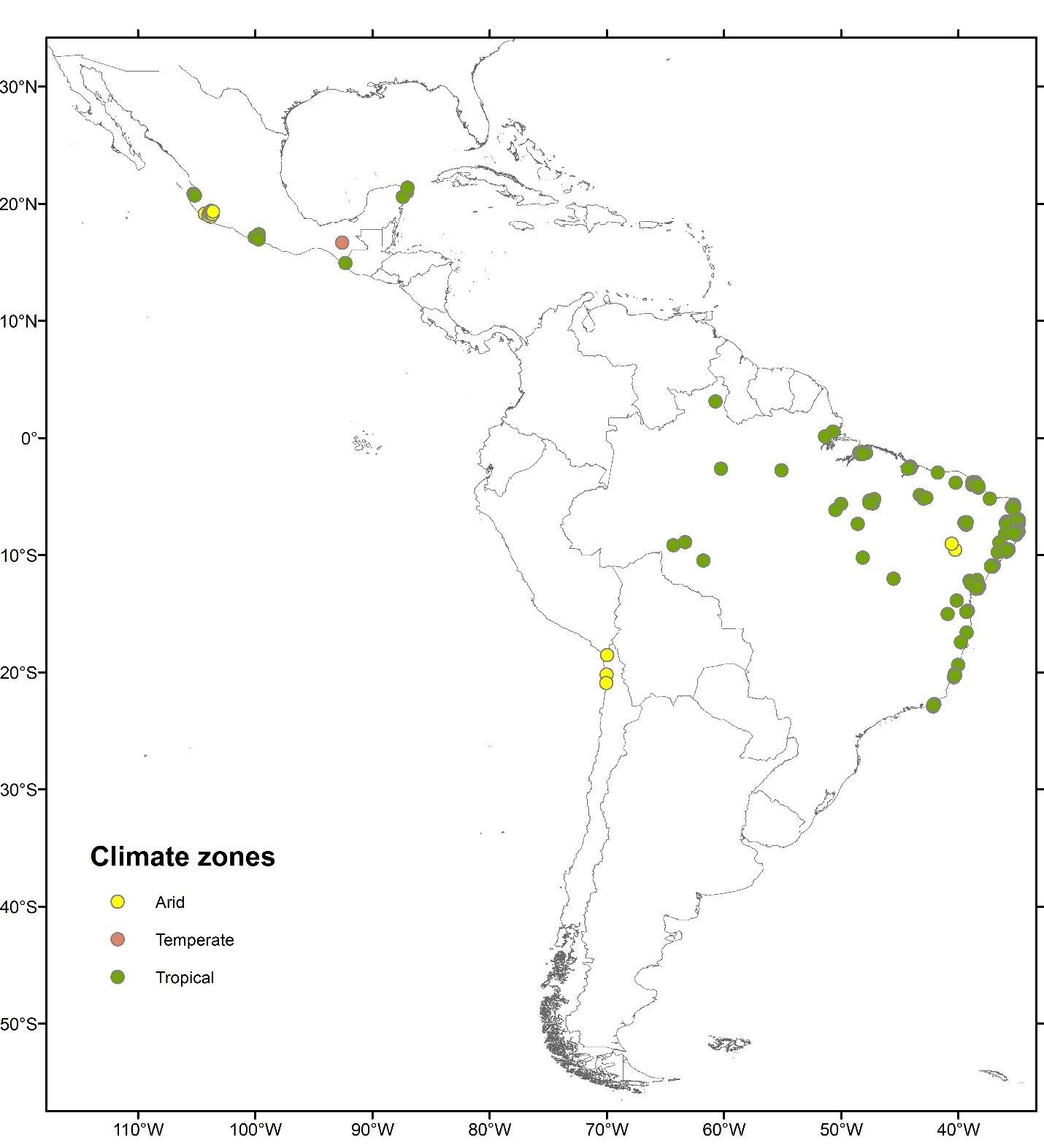


Figure S22. Sub-cities with annual temperature variation ≤ 10 °C and their corresponding climate zones


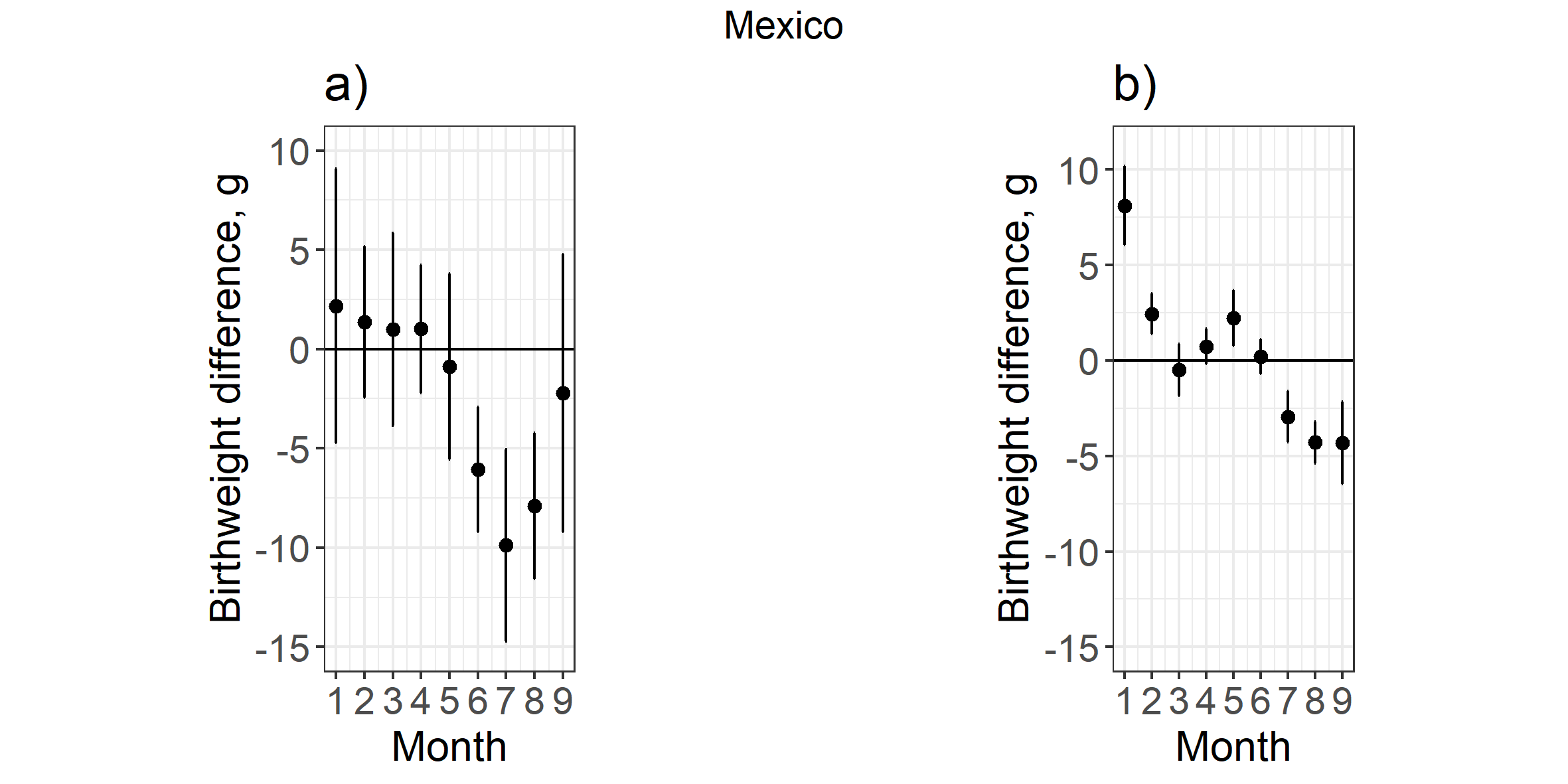

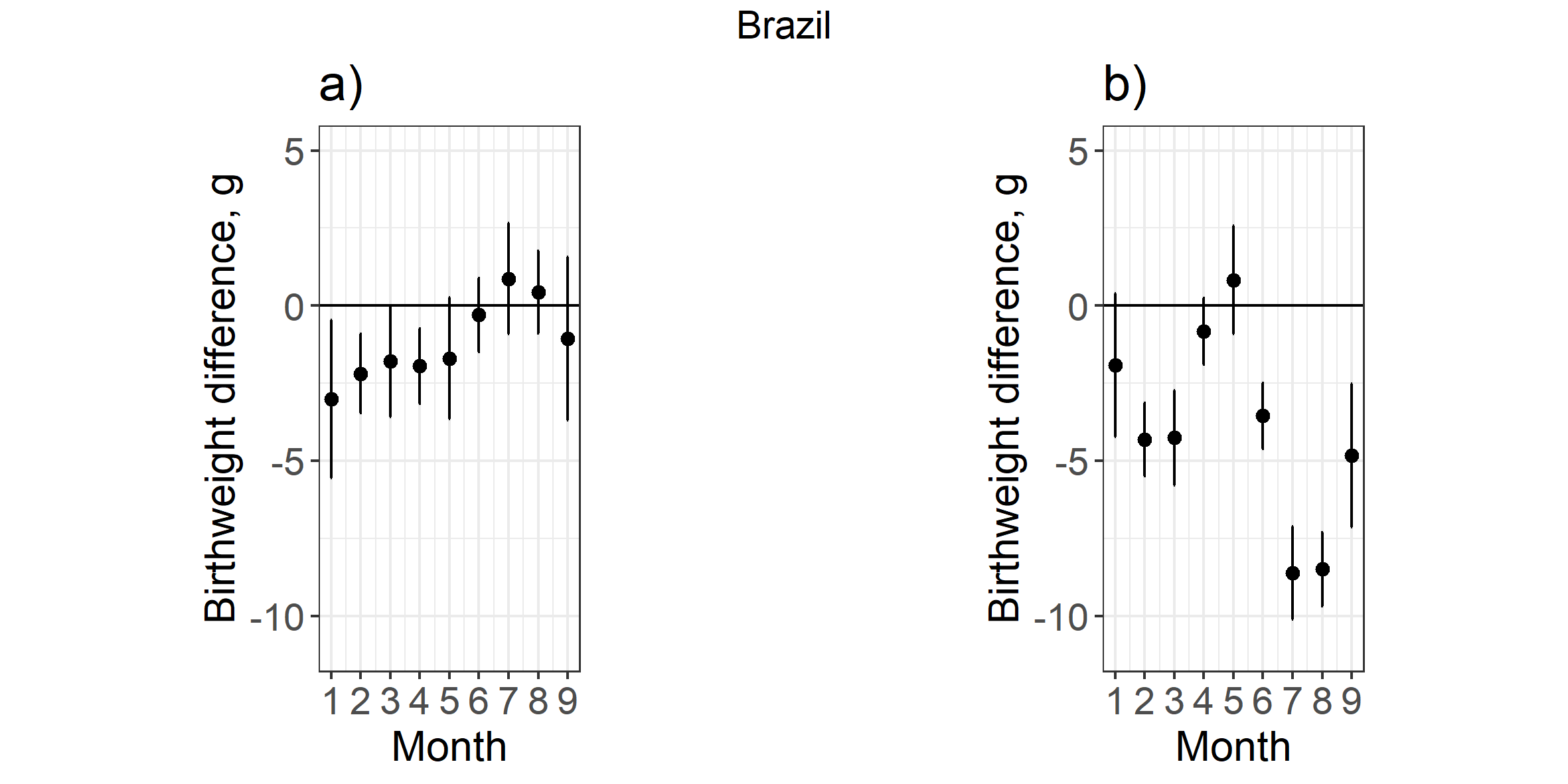

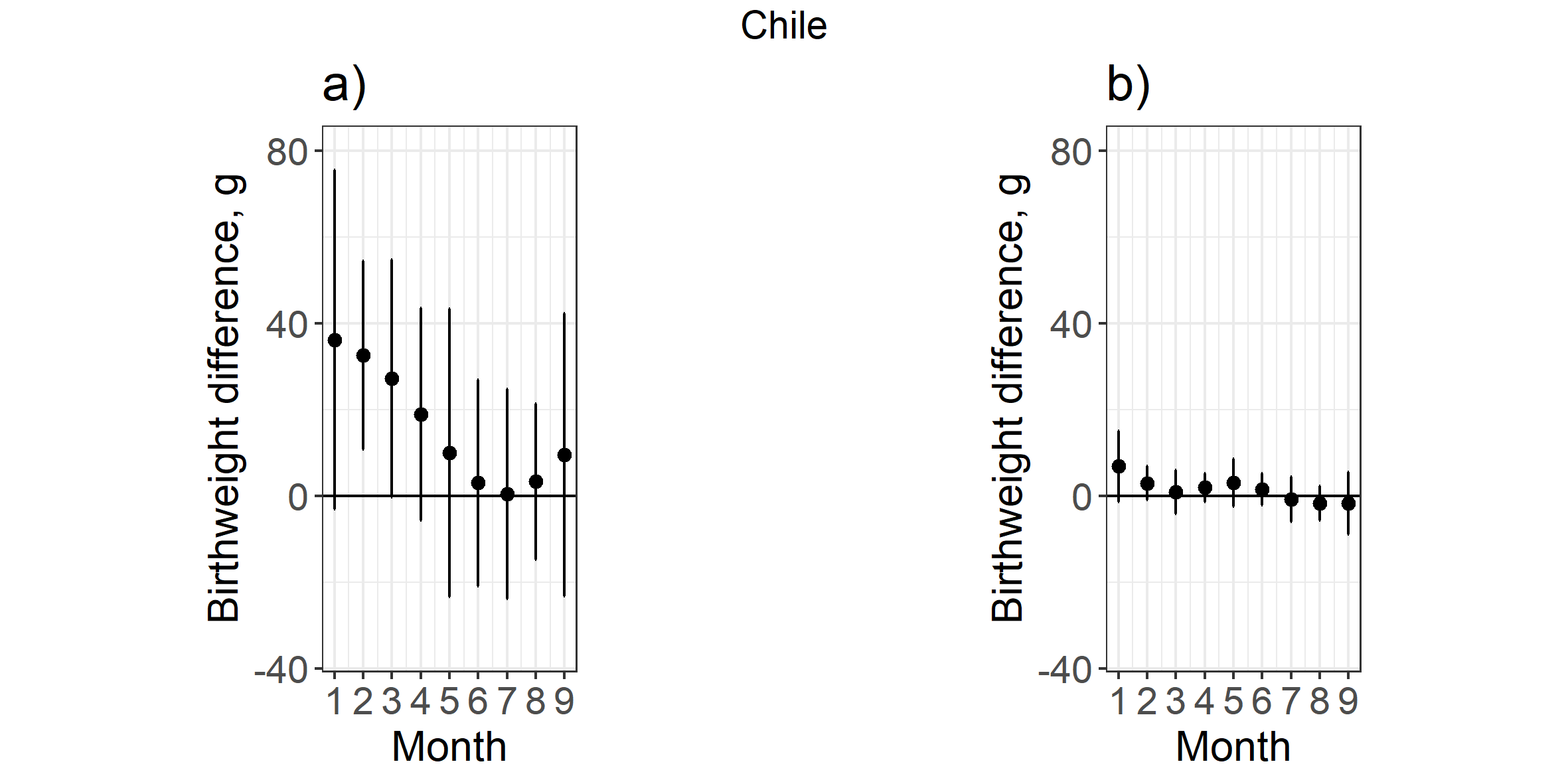


Figure S23. Difference in birthweight associated with a 5°C higher temperature in each month of gestation, relative to the average monthly temperature in the two categories of sub-cities by temperature range for every country:

a. sub-cities with limited temperature range (≤ 10°C)

b. sub-cities with temperature range > 10°C)

The estimates are obtained from the distributed lag non-linear models stratified by live births from sub-cities in the two groups of temperature range, adjusted for child sex, mother’s age, education, partnership status, whether the mother had previous births, calendar year of child’s birth, season of conception, and include a random intercept for the sub-city of mother’s residence at the time of the child’s birth. Estimates for every exposure window account for temperature exposure during all the other exposure windows during the gestational period.

**References**

1. Muñoz Sabater J. ERA5-Land hourly data from 1981 to present, Copernicus Climate Change Service (C3S) Climate Data Store (CDS). 2019.

2. Hersbach H, Bell B, Berrisford P, et al. ERA5 hourly data on single levels from 1979 to present. *Copernicus Climate Change Service (C3S) Climate Data Store (CDS)*. 2018;10

3. Gasparrini A, Armstrong B, Kenward MG. Distributed lag non‐linear models. *Statistics in medicine*. 2010;29(21):2224-2234.

4. Shah PS, births KSGoDoLP. Parity and low birth weight and preterm birth: a systematic review and meta‐analyses. *Acta obstetricia et gynecologica Scandinavica*. 2010;89(7):862-875.
